# Under ice plankton and lipid dynamics in a subarctic lake

**DOI:** 10.1093/plankt/fbae018

**Published:** 2024-05-03

**Authors:** Erwin Kers, Eva Leu, Per-Arne Amundsen, Raul Primicerio, Martin Kainz, Amanda E Poste

**Affiliations:** Department of Arctic and Marine Biology, UiT The Arctic University of Norway, Framstredet 39, Tromsø 9037, Norway; Fram Centre, Akvaplan-niva, Hjalmar Johansensgate 14, Tromsø 9007, Norway; Department of Arctic and Marine Biology, UiT The Arctic University of Norway, Framstredet 39, Tromsø 9037, Norway; Department of Arctic and Marine Biology, UiT The Arctic University of Norway, Framstredet 39, Tromsø 9037, Norway; WasserCluster Lunz - Biologische Station, Dr. Carl Kupelwieser Promenade 5, Lunz am See 3293, Austria; Research Lab for Aquatic Ecosystem Research and Health, Danube University Krems, Dr. Karl Dorrek Straße 30, Krems 3500, Austria; Department of Arctic and Marine Biology, UiT The Arctic University of Norway, Framstredet 39, Tromsø 9037, Norway; Norwegian Institute for Water Research, Økernveien 94, Oslo 0579, Norway; Norwegian Institute for Nature Research, Hjalmar Johansensgate 14, Tromsø 9007, Norway

**Keywords:** winter ecology, lake ice, Arctic, fatty acids, zooplankton, copepods, phytoplankton

## Abstract

Climate warming causes shorter winters and changes in ice and snow cover in subarctic lakes, highlighting the need to better understand under-ice ecosystem functioning. The plankton community in a subarctic, oligotrophic lake was studied throughout the ice-covered season, focusing on lipid dynamics and life history traits in two actively overwintering copepods, *Cyclops scutifer* and *Eudiaptomus graciloides.* Whereas *C. scutifer* was overwintering in C-IV to C-V stage, *E. graciloides* reproduced under ice cover. Both species had accumulated lipids prior to ice-on and showed a substantial decrease in total lipid content throughout the ice-covered period: *E. graciloides* (60%–38% dw) and *C. scutifer* (73%–33% dw). Polyunsaturated fatty acids of algal origin were highest in *E. graciloides* and declined strongly in both species. Stearidonic acid (18:4n-3) content in *E. graciloides* was particularly high and decreased rapidly during the study period by 50%, probably due to reproduction. The copepods differed in feeding behavior, with the omnivore *C. scutifer* continuing to accumulate lipids until January, whereas the herbivorous *E. graciloides* accumulated lipids from under-ice primary production during the last months of ice-cover. Our findings emphasize the importance of lipid accumulation and utilization for actively overwintering copepods irrespective of the timing of their reproduction.

## INTRODUCTION

Winter, here defined as the ice-covered period of a lake, is an essential but traditionally understudied season in limnology (McMeans *et al.*, 2020). The winter season has been assumed to be a period of low biological activity. This, combined with the practical difficulties in conducting studies under the ice (Block *et al.*, 2019), has resulted in ecological research mainly being focused on the more productive summer period. Considering that half of the world’s lakes (equaling more than 50 million lakes; Verpoorter *et al.*, 2014) freeze periodically (Hampton *et al.*, 2017), the need of a better understanding of the winter season is obvious, not the least as processes during winter set the stage for the following productive season (Dokulil *et al.*, 2014; O’Reilly *et al.*, 2015). In recent decades, there has been an increased focus on winter processes in lakes, especially related to impacts of climate change (Benson *et al.*, 2012; Wang *et al.*, 2012; Leppäranta, 2015; Filazzola *et al.*, 2020; Magnuson *et al.*, 2000; Ozersky *et al.*, 2021), which is expected to force major changes in lake ecology (Hobbie *et al.*, 1999). Yet, high-latitude lakes, which can be ice-covered for more than half of the year, are still poorly studied.

Lake water temperatures have been rising globally for several decades due to climate change, displaying particularly rapid trends in Northern Europe (Schneider and Hook, 2010; O’Reilly *et al.*, 2015). Ice-cover duration of lakes has been decreasing (Magnuson *et al.*, 2000; Kainz *et al.*, 2017) and, in northern Fennoscandia, it is projected to decline further by 3–14 weeks by 2100 (Gebre *et al.*, 2014). Timing and duration of ice and snow cover have important implications for several essential biological processes in lakes, by limiting incoming irradiance and affecting mixing and stratification patterns (Moore *et al.*, 2009; Salonen *et al.*, 2009; Benson *et al.*, 2012; Ozersky *et al.*, 2021). Snow and ice conditions influence under-ice light availability in lakes. Expected changes to lake ice and snow cover due to climate change include later ice-on, earlier snow melt/ice-off and increased frequency of rain on snow events (Cohen *et al.*, 2015), all of which may increase light availability (Leppäranta, 2015), although this will be moderated by regional changes in snow accumulation. The Arctic is characterized by low-light conditions (polar night) during late autumn and midwinter, i.e. during early ice cover, and high-light intensities (midnight sun) at the end of the ice-cover period (Berge *et al.*, 2015). Knowledge on the relation between ice (and snow) quality, primary production and resource availability is currently lacking even though this is vital to understand the ecology of seasonally ice-covered lakes (Jansen *et al.*, 2021).

Life under lake ice must adjust to low-light conditions (Bolsenga *et al.*, 1991), low temperatures (Raven and Geider, 1988), lack of mixing and the absence of oxygen exchange at the surface (Kirillin *et al.*, 2012). A study of seasonally ice-covered lakes around the world showed that phytoplankton is generally far less abundant in winter compared to summer [43% chlorophyll-*a* (chl-*a*), 15% of phytoplankton biovolume], and zooplankton abundances are similar only about 25% of the summer concentrations (Hampton *et al.*, 2017). In addition to plankton densities, the nutritional content (e.g. lipid composition) of plankton communities differs between summer and winter. However, there is currently a general lack of lipid data from the ice-covered season (Fernandes and McMeans, 2019). In particular, except for a few noteworthy contributions (e.g. Syväranta and Rautio, 2010; Mariash *et al.*, 2016; Grosbois *et al.*, 2017), there is a shortage of studies describing how lipid content and composition of phytoplankton and zooplankton change over the course of the ice-covered season (Perga *et al.*, 2020).

Copepods, and, less frequently cladocerans, have been observed to grow and reproduce under the ice (Rautio *et al.*, 2011; Rigler *et al.*, 1974). During ice-cover, copepods often dominate the zooplankton community (Perga *et al.*, 2020; Shchapov *et al.*, 2021; Shchapov and Ozersky, 2023). To cope with the harsher winter conditions at high latitudes, actively overwintering zooplankton can use previously acquired lipids (Grosbois *et al.*, 2017), in addition to the limited food sources available under the ice (i.e. heterotrophs, bacteria, phototrophs) (Karlsson and Säwström, 2009; Rautio *et al.*, 2011; Säwström *et al.*, 2009). Rotifers are present under ice cover although in lower abundance compared to summer (Jensen, 2019; Primicerio and Klemetsen, 1999). Many species (especially copepods) accumulate large lipid reserves during the growing season to prepare for these periods of lower productivity (Kuosa and Gyllenberg, 1989; Goulden *et al.*, 1999). In marine zooplankton species, accumulation of substantial lipid reserves (often as wax esters) has also been reported (Lee *et al.*, 2006).

Actively overwintering copepods have different life history strategies and phenologies. Calanoid copepods can reproduce under ice cover (Pasternak, 1999), whereas cyclopoid copepods have been observed to stay active in the C-IV stage (Elgmork and Eie, 1989) and reproduce in July (Primicerio and Klemetsen, 1999). The cyclopoid *Cyclops scutifer* is omnivorous, although mainly herbivorous in the earlier life stages and has even been successfully bred on an algal diet (Pejler, 1983). Many adult cyclopoids are known to consume small heterotrophic prey such as rotifers and nauplii (Langeland and Reinertsen, 1982), which can also provide a trophic pathway to bacterial production via the microbial loop (Kalinowska *et al.*, 2015). In contrast, the herbivorous calanoid copepod *Eudiaptomus graciloides* cannot easily make use of additional food resources, such as rotifers and nauplii, which might be more readily available during winter. However, there is evidence of marine zooplankton shifting from herbivory to omnivory during polar night, even for taxa that are considered to be primarily herbivorous (Kunisch *et al.*, 2023).

Especially for herbivorous zooplankton, light limitation in ice-covered lakes implies that autotrophic phytoplankton are only available in very low quantities, and previously acquired lipids may thus play a vital role in the survival of zooplankton during long periods of ice cover (Grosbois *et al.*, 2017; Perga *et al.*, 2020). In subarctic lakes, lipid content of calanoid and cyclopoid copepods has been observed to differ by 20–60% between seasons, with the highest levels observed in early winter (Syväranta and Rautio, 2010). Grosbois *et al.* (2017) report lipid content reaching 76% of total body mass for copepods at the onset of ice-cover in a Canadian lake.

Multiple factors influence zooplankton lipid content and composition, including food availability and quality and, to a lesser extent, physical conditions. The nutritional quality, and particularly the lipid composition, of available food sources is important for survival, somatic growth and reproduction of consumers and their predators. In particular, the contribution of polyunsaturated fatty acids (PUFA) to the total lipid pool at the base of the food web (i.e. in primary and bacterial producers) and in zooplankton is of ecophysiological importance for zooplankton. Dietary PUFA can improve zooplankton growth rates (Gulati and Demott, 1997) and is transferred to consumers at higher trophic levels, such as fish and, eventually, humans. The phytoplankton community composition and lake trophic status (i.e. nutrient concentrations) are key predictors of lipid composition and content in aquatic food webs (Taipale *et al.*, 2013, 2016). Temperature is another important factor potentially influencing lipid composition, with higher PUFA contents at lower temperatures (Farkas and Herodek, 1964; Farkas, 1984; Masclaux *et al.*, 2012; McMeans *et al.*, 2015), which in turn are important for maintaining fluidity in biological membranes (Pilecky *et al.*, 2021). In addition, various aspects of life history traits can have implications for copepod lipid composition, such as overwintering and reproduction strategies (Hartwich *et al.*, 2013). However, little is known about many of these processes under ice-covered conditions.

Taken together, climate driven changes in ice-cover duration, shifts in plankton community structure and changing environmental conditions under-ice have the potential to lead to changes in lipid availability, food web composition and diet transfer, with a broad range of consequences for aquatic ecosystem functioning. This highlights the need for information on plankton dynamics under ice cover to understand and predict future ecosystem responses to changing ice- and snow-cover in high-latitude lakes (Salonen *et al.*, 2009). The aim of this study was to investigate the acquisition, compositional change and utilization of zooplankton lipids as a function of environmental conditions, community structure and seasonal development during the ice-covered period of a subarctic lake. We assessed differences in the seasonal changes of lipid profiles of two actively overwintering copepod species with broad circumarctic distribution (*C. scutifer* and *E. graciloides*) that have contrasting life history strategies and timing of reproduction. We hypothesized; (i) decreasing lipid content of both species during winter with distinctly higher lipid contents at the beginning compared to the end of the ice-cover period and (ii) a more pronounced reduction of lipids in *E. graciloides* than in *C. scutifer* due to reproduction during ice cover. We expected both copepods (especially the omnivorous *C. scutifer*) to rely more on non-phytoplankton food sources during winter due to limited primary production under ice cover.

## METHOD

### Sampling site

Samples were collected from lake Takvatn (N 69.09, E 19.14) (Supplementary material Fig. S1) on an approximately monthly basis during the ice-covered period of 2020–2021, including additional sampling prior to and after the onset of ice cover. Lake Takvatn is an oligotrophic, dimictic lake (214 m a.s.l.) in northern Norway with a surface area of 15 km^2^ and a maximum depth of 80 m. The sampling station of the present study had a depth of 60 m and was located in the southeast part of the lake (Fig. S1). The lake typically freezes in late November, and ice-off often occurs in early June (Primicerio, 2000). In 2020, ice-on occurred in the first week of December and ice-off in the first week of June 2021. The polar night (i.e. the period when the sun is consistently below the horizon) lasts from late November until mid-January, while the region experiences 24-h of daylight from mid-May until mid-July.

### Field observations

Field sampling included measurements of snow depth, ice thickness, Secchi depth and light intensity (starting in February). Vertical profiles were carried out from 0 to 60 m using a CTD (SAIV SD-204), providing data on conductivity, temperature, depth, oxygen and *in situ* chlorophyll fluorescence. Light measurements of integrated photosynthetically active radiation (PAR, 400–700 nm) were performed using a cosine-corrected sensor (LICOR LI-190/R, LI-192) measuring above and below the ice, usually close to noon. For the light transmittance measurements, the sensor was mounted on an L-arm, and measurements were first taken underneath undisturbed snow and then repeated after snow removal to quantify the contribution of snow to the overall changes in transmittance. In addition, a light logger (DEFI2-L) was deployed under the ice measuring PAR at 15-min intervals from 5 February 2021 to 8 June 2021.

### Water sampling and sample processing

Discrete water samples were taken just below the ice (0 m) and at 58m depth using a Ruttner water sampler. An integrated water sample was taken from below the water surface (0–10 m; during open water periods) or below the bottom of the ice. The integrated water samples were pooled, transferred to black 5 L jugs, and kept dark and cool until further processing. Separate samples were taken from just below the ice using a pump and hose to obtain water from the uppermost layer just below the bottom of the ice.

For integrated (0–10 m) and bottom (58 m) water samples, a subsample (100 mL) was taken for the analysis of SiO_2_, NO_2_/NO_3_, PO_4_ and NH_4_. The sample was filtered (Whatman 0.7 μm GF/F filter) using a syringe and collected into acid-washed plastic bottles that were pre-rinsed and then filled with the filtrate and subsequently acidified with 4 N H_2_SO_4_ to a concentration of 1% by volume.

Subsamples from water samples (0–10 m, surface and bottom water) were filtered through glass fiber filters (Whatman GF/F; nominal pore size 0.7 μm) for chl-*a* analysis. Particulate organic matter (POM; from 0–10 m) for total lipids and fatty acid (FA) analysis was filtered through pre-combusted GF/F filters (combusted at 450°C for 4 h). The volume of the filtered water ranged from 2 to 5 L, dependent on the color of the filters after filtration: i.e. when little color was visible on the filter (indicating low biomass) more water was filtered to obtain enough biomass for the analysis. All filters were kept frozen at −20°C except for the samples for FA analysis which were stored at −80°C to limit lipolytic degradation.

### Nutrient and chl-*a* analysis

Nutrient analyses were performed at the Norwegian Institute for Water Research (NIVA, Oslo, Norway) using standard accredited methods (as described in Braaten *et al.*, 2020). Chl-*a* concentrations were determined fluorometrically for the upper lake layer (0–10 m) and surface samples at Akvaplan-niva (Tromsø, Norway) (Parsons *et al.*, 1984). Filters were extracted in 90% acetone in the dark at −20°C for 12 h, subsequently an extract (3–4 mL) was transferred to a fluorometer cuvette and measured using a Turner 10-AU-000 fluorometer.

### Phytoplankton sampling and taxonomic analyses

For microscopic identification, phytoplankton samples were collected from the upper lake layer (0–10 m). Samples were preserved using 1% v/v Lugol’s iodine and stored in the dark until further analysis. For taxonomic analysis, samples were well-mixed and a 50 mL subsample was transferred to an Utermöhl settling chamber and left for 24 h. After settling, the sample was counted using an inverted microscope. Phytoplankton (minimum of 200 cells per sample) were identified to class level.

### Zooplankton sampling and taxonomic analyses

On each sampling date, zooplankton samples were collected with a 90 μm plankton net from 0–60 and 0–20 m. In addition to these two samples, a larger pooled sample for sorting to species level was collected by combining material from three net hauls from 0–60 m. Rotifer samples were taken from 30–0 m with a 50 μm plankton net, these samples were collected in conjunction with a longer time series based on depth interval, thus the difference in depth between zooplankton and rotifer samples. All samples were placed in a dark cooler at the site, until transferred to a refrigerator in the lab, ~3–4 h after sample collection.

On the same day as sampling, zooplankton samples were split into subsamples containing a known fraction of the total sample using a plankton splitter. Subsamples were taken for microscopic identification, quantification of biomass and FA analysis, with subsample size dependent on ensuring sufficient biomass for FA analysis. Zooplankton subsamples for microscopic identification were preserved in 10% formalin (by volume) and were stored at room temperature. After rinsing out the formalin with water, the species were identified and zooplankton samples counted to stage (Nauplii, C-I to C-III, C-IV to C-V, adult male, adult female and egg-bearing) under a stereomicroscope and then were placed in ethanol for longer term storage. Rotifer samples were also preserved in 10% formalin (by volume) and after rinsing identified under a stereomicroscope. Zooplankton biomass was measured by filtering a known fraction of the net haul through a pre-weighed QMA (Whatman™ quartz fiber) filter that was then dried at 60°C overnight and then re-weighed.

The pooled zooplankton from the 90 μm net haul was stored at 5°C in the dark until sorting to species-level the day after sampling, when *C. scutifer* and *E. graciloides* were separated using a stereo microscope at ×40 magnification. Living zooplankton were placed in small droplets on a glass petri-dish at room temperature using a glass pipet and subsequently transferred to cryovials (placed on ice), each consisting of about 200 individuals per species, including all stages except nauplii. Three subsamples were taken for each species and stored at −80°C until FA analysis. Samples for FA analysis were freeze-dried before being transported to WasserCluster Lunz, Austria, for lipid analysis.

### Total lipid and fatty acid analysis

Lipids and their FA were analyzed using established methods as reported elsewhere (Heissenberger *et al.*, 2010). In brief, freeze-dried zooplankton and filters were extracted using chloroform:methanol (2:1, vol/vol) followed by sonication, vortexing and centrifuging 3 times to remove non-lipid materials. Pooled organic phases were evaporated to a final volume (1.5 mL) under N_2_. Total lipid contents were obtained by injecting aliquots (100 μL) of the total lipid extract into pre-weighed tin capsules, which were weighed again after the liquid extract had evaporated. For fatty acid methyl esters (FAME) formation, a known volume of lipid extracts were incubated with sulfuric acid:methanol (1:100 vol/vol) for 16 h at 50°C, following the addition of KHCO_3_ and hexane. Samples were shaken, vortexed and centrifuged and the upper organic layers collected 2 times, pooled and concentrated under N_2_.

FAME were analyzed using a gas chromatograph [TRACE GC THERMO, detector: FID 260°C, carrier gas: He: 1 mL/min, detector gases: H_2_: 40 mL/min, N_2_: 45 mL/min, air: 450 mL/min, temperature ramp: 140°C (5 min)—4°C/min—240°C (20 min) = (50 min)] equipped with a temperature-programmable injector and an autosampler. A Supelco™ SP-2560 column (100 m, 25 mm i.d., 0.2 μm film thickness) was used for FAME separation. Comparison of the retention times with standards led to identification of FAME (37-component FAME Mix, 47 885-U, Supelco; Sigma-Aldrich, Bellefonte, Pennsylvania). Chromeleon 7™ was used for peak integration and concentrations of FA were calculated based on individual calibration curves. Results were reported as mass fractions (e.g. mg FAME g dw^−1^) or as mass percentages (%).

### Data analysis

Data visualization and statistical analyses were carried out in R version R-4.0.3 (R Core Team, 2021). All lipid-related data from samples obtained on 27 October 2020 have been removed from further analyses. These were the first samples sorted for FA analysis and took considerably longer to process, which caused samples to warm up. Therefore, the accuracy of these samples cannot be guaranteed since the lipids might have been oxidized during sorting. For POM, we were not able to obtain data on dry weight due to the very low biomass on the GF/F filters (i.e. weighed filters would have large uncertainty), and as such, FA in POM were reported per unit water volume (i.e. μg FA L^−1^ in lake water).

Outliers in the CTD data (due to sampling error) were removed, and only the downcast was selected. The multilevel B-splines interpolation method was used for plotting the CTD data (Lee *et al.*, 1997), using the MBA package in R (Finley *et al.*, 2017).

Changes in lipid content and composition over time were assessed by local polynomial regression to estimate the trend of total lipids, using the GGplot2 package in R (Wickham, 2016). Fatty acids were grouped as biomarkers for bacterial, terrestrial and bio-indicators for the sum of phytoplankton FA (Kainz *et al.*, 2003; Kainz and Mazumder, 2005; Grosbois *et al.*, 2017) (individual FA included in each biomarker group are summarized in Table I). We chose to use total PUFA as an indicator for phytoplankton since this is often a good indicator for contribution of algal FA, although it should be noted that some PUFA are also known to have non-algal origins.

**Table I:** *Grouped fatty acids used as biomarkers with the individual fatty acids they contain. Bacterial FA biomarkers were selected based on*  *Kainz* et al. *(2003)**. Terrestrial FA biomarkers were selected based on*  *Grosbois* et al. *(2017)*

Grouped	Individual fatty acids
Bacterial fatty acid biomarkers	iso-15:0, anteiso-15:0, C15:0, iso-16:0, iso-17:0, C17:0, 9,10D16, C18:1n-7, C18:1n-6, 9,10D18
Phytoplankton biomarkers	Sum of PUFAs
Terrestrial fatty acid biomarkers	C20:0, C22:0, C23:0, C24:0

To visualize seasonal and interspecies differences in the FA profiles of *C. scutifer* and *E. graciloides*, a correspondence analysis (CA) was performed on FA data using the *EasyCoda* (Greenacre, 2018) package in R. The following FA were removed from the analysis because they could not be detected in the samples: 9,10Δ16 and 20:1n-9; the internal standard (19:0) was also removed. FA labels were shown in the plot when they contributed more to the inertia than expected if all FA were equal; eicosapentaenoic acid (20:5n-3, EPA) was added manually to evaluate the potential contribution of this important PUFA. To assess the variability in FA composition attributable to species and sampling date, a canonical correspondence analysis (CCA) was applied. The same FA were omitted from the CCA as for the CA. C20:5n-3 was added manually since this is known to be an important FA. The significance of the CCA model and of the marginal effects of the predictors were tested by permutation analysis. The CCA implementation and the permutation tests were performed using the *vegan* package in R (Oksanen *et al.*, 2007).

## RESULTS

### Environmental conditions

**Fig. 1:**
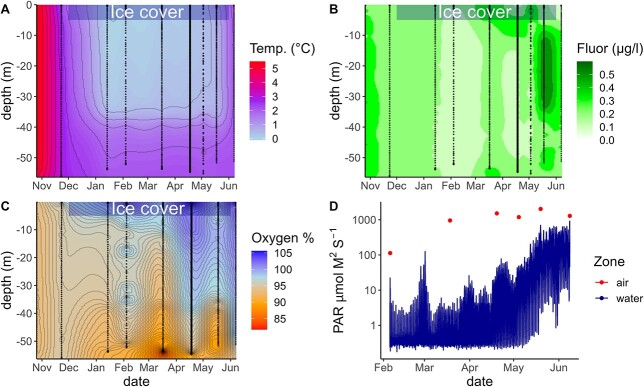
Data from CTD profiles (**A**) Temperature, (**B**) Chlorophyll fluorescence and (**C**) Oxygen saturation and (**D**) the moored light sensor measuring PAR. CTD data have been interpolated over the study period (dotted lines indicate actual observations from sampling dates), while for light measurements, the line shows continuous light measurements directly below ice while dots show light measurements directly above ice.

In October and November, Takvatn was ice-free and well mixed (Fig. 1) with water temperatures of approximately 5 and 3°C, respectively, throughout the entire water column. The lake was stratified during ice-cover between January and May, and mixed in June after ice-off (Fig. 1A). From mid-January to mid-May water temperatures ranged from <1°C in the upper 35 m to 2°C below 35 m. Chl-*a* (based on CTD measurements) concentrations were the lowest in February (0.03 μg L^−1^) and increased in April and May in the upper 10 m (0.3 μg L^−1^). Fluorescence was the highest in late May (0.5 μg L^−1^) (Fig. 1B).

Lake Takvatn was ice-covered from the beginning of December until the beginning of June. Unusual for this lake, December and January were characterized by clear ice without snow. Some snowfall occurred in February and the snow depth was 4.5 cm on the sampling date in February. Ice thickness was highest in March at 73 cm, with a snow depth of 21 cm (Table S1). In March, dry snow reduced the transmittance to 1% of the values measured in air, and when snow was removed, the light transmittance was 19.5%. In April and May, there was a layer of slush snow on top of the ice. The snow layer had disappeared by May 19, and only ice (with a frozen slush layer) remained until ice-off. Underwater light increased rapidly throughout May (up to 600 μmol m^2^ s^−1^) and into June, in contrast to April when light never exceeded 50 μmol m^2^ s^−1^. The June measurement was carried out under overcast conditions, and therefore yielding lower irradiance values than would have been measured on a clear day (Fig. 1D).

**Fig. 2:**
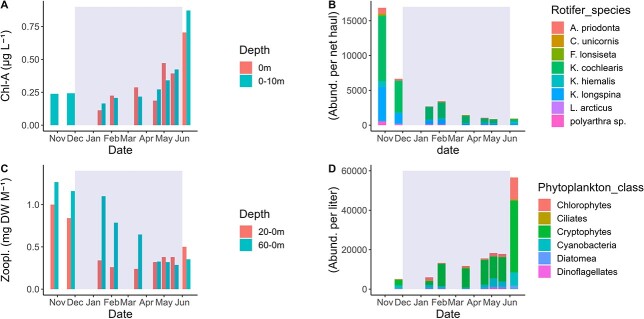
(**A**) Chlorophyll-a concentrations in integrated 0–10 m samples and directly below the ice (0 m)., In October and November, no surface samples were taken since there was no ice cover and the lake surface waters were well mixed. (**B**) Rotifer species composition (full species names; *Keratella cochlearis*, Kellicotta longspina, *Keratella hiemalis*, Asplancha priodonta, *Lepidurus arcticus*, Filinia lonsiseta, *Conochilus unicornis*). (**C**) Zooplankton biomass (including rotifers) for samples collected from 0–20 to 0–60 meters, biomass is reported as the dry weight per vertical meter hauled. (**D**) Phytoplankton (n cells per liter) for samples collected from 10–0 m. Gray shaded areas in all plots indicate the ice-covered period.

Oxygen saturation was high (above 85%) in most of the water column except for March below 50 m (Fig. 1C). Oxygen saturation increased from April to June to levels above 100% corresponding with the increase in fluorescence in the upper water column. Nitrite and nitrate concentrations increased slightly during ice cover from 3.35 to 4.43 μM at the end of ice cover (Table S2). Deep water (58 m) showed similar nutrient levels compared to the surface water (0–10 m), although from March–June nitrate and nitrite (and to a lesser degree silicate) concentrations were lower in surface water than at 58 m. Chl-*a* concentrations were measurable throughout the winter with only minor differences between the top 10 m layer and immediately below the ice (0 m) (0.01–0.15 μg L^−1^). There was an increase in chl-*a* concentrations towards May–June (Fig. 2A).

### Plankton dynamics

**Fig. 3:**
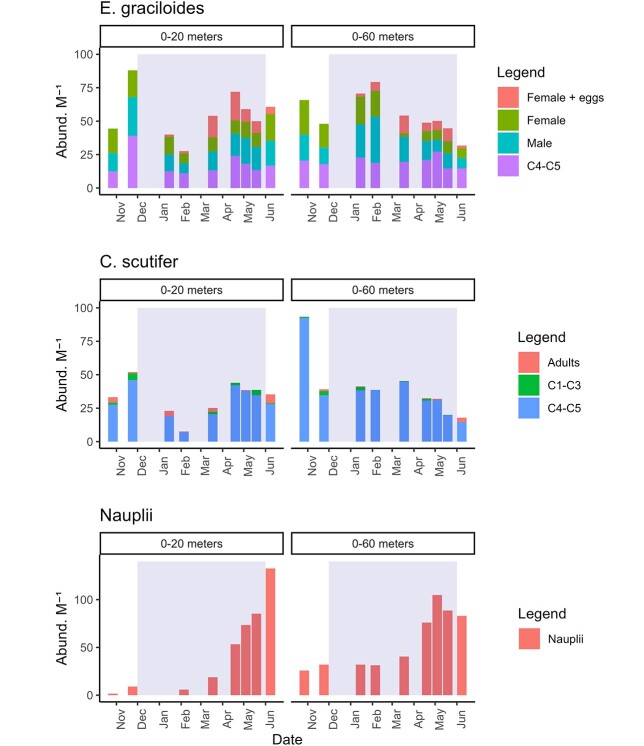
The abundance of *Eudiaptomus graciloides* (top), *Cyclops scutifer* (middle) and nauplii (bottom) from 0–20 to 0–60 m. Abundances are reported as number of individuals per vertical meter hauled, to facilitate comparison between 0–20 and 0–60 m hauls. For copepodite and adult stages (top and middle plots), abundances are reported by development stage. For *E. graciloides* it was possible to further distinguish between male adults, females carrying and egg sack (“Female + eggs”) and females without eggs (“Female”). Gray shaded areas indicate the ice-covered period.

Despite the prevalent assumption that zooplankton tend to have low abundance under ice, in the current study, zooplankton abundances (0–60 m) during the early ice cover period were not substantially lower than during late autumn before ice-on (October–November) (Fig. 2C, Fig. 3). Phytoplankton was present during the entire ice-covered period, with cryptophytes being most dominant in abundance (between 1100–29 000 individuals L^−1^). Chlorophytes and cyanobacteria increased (from 200 to 2083 individuals L^−1^ and from 40 to 1301 individuals L^−1^, respectively) from April to June (Fig. 2D). Dinoflagellates, chlorophytes and cyanobacteria were notably higher in samples collected at 0 m compared to 0–10 m from April to June (Fig. S2). Due to large size differences between the classes these abundance data were not a good representation of the relative biomass contribution of the various phytoplankton classes observed.

Zooplankton biomass (normalized to haul depth; in mg m^−1^ hauled) declined during the ice-covered period (Fig. 2C). When comparing the zooplankton abundances of ice free (October and November) to the early ice-covered time period there is not a large decline in the 0–60 m depth (Fig. 2C, Fig. 3). From January to March there was more biomass per vertical meter from 0–60 m in the water column compared to the upper 20 m of the water column (Fig. 2C). In April, there was an increase in zooplankton biomass in the upper 20 m compared to the 0–60 m, with a slight increase in June. From January to February the highest abundance of zooplankton was recorded below 20 m, which was also illustrated by the zooplankton biomass (Fig. 2C). Rotifers were present all winter, but their abundance declined with the largest decrease from October to November (16 853 to 6656 N per net haul). *Keratella cochlearis* was the most abundant rotifer (Fig. 2B). Under ice cover the overall rotifer abundance slowly declined.

**Fig. 4:**
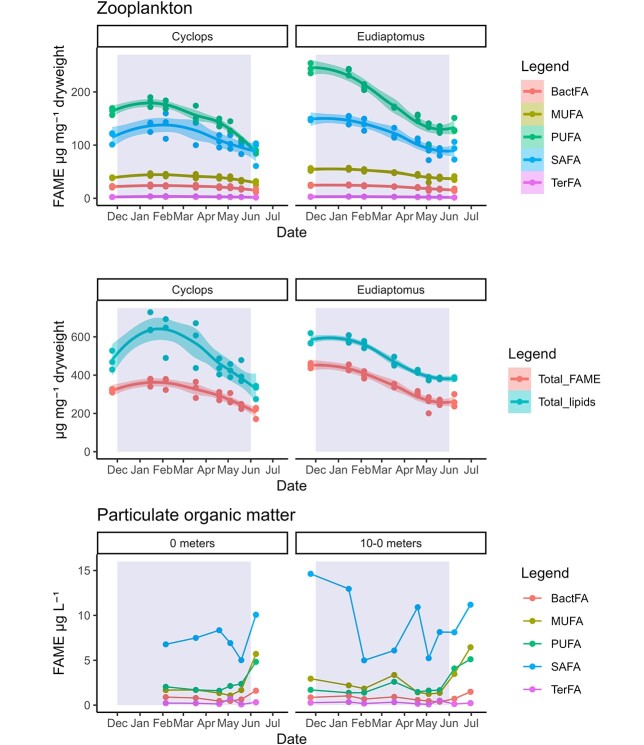
Fatty acid content for biomarker groups for zooplankton and POM. Y-axis shows the fatty acids in μg mg^−1^ dry weight for zooplankton and μg L^−1^ for POM (seston). BactFA, Bacterial fatty acid biomarkers; MUFA, monounsaturated fatty acids; PUFA, polyunsaturated fatty acids; SAFA, saturated fatty acids; TerFA, terrestrial fatty acid biomarkers. The line is a smoother using the loess method, the confidence interval (95%) is visualized by the light-colored area around the lines.

*E. graciloides* and *C. scutifer* dominated the large zooplankton during ice cover with very few other taxa detected, their abundance declined during the ice-covered period. The decline in zooplankton biomass and abundance corresponded with a decrease in lipid content (Figs 3 and 4). For *E. graciloides*, some females carrying an egg sac were observed as early as January. The number of females with eggs increased strongly in February and March (Fig. 3). Males were present, and spermatophores attached to females were occasionally observed in the same period. The C-IV to C-V stages were also present during ice cover, and occasionally C-I to C-III stages were observed, but only sporadically and with negligible abundances. Both species were observed with brightly orange-colored lipid droplets during winter.

*C. scutifer* was mainly present in copepodite stages C-IV and C-V stage throughout the winter. Very small numbers of adults and C-I to C-III were observed. The C-IV to C-V stages decreased throughout the ice-covered period in the 60 m net hauls, a trend less pronounced in the 20 m hauls, with the lowest numbers observed in January and February. Nauplii occurred in large numbers with an increase in March when *E. graciloides* started to release eggs. Nauplii were most abundant in the upper 20 m, with our results suggesting lower abundances in the deeper part of the water column.

### Lipid dynamics and fatty acid composition

The total lipid content decreased during the ice-covered period for both *E. graciloides* (from 60% to 38% of dw) and *C. scutifer* (from 73% to 33% of dw). The content of all FA groups (i.e. saturated, SAFA; monounsaturated, MUFA and PUFA) declined during the ice-cover period in both species, although the lipid and FA content stabilized in *E. graciloides* during May and June (Fig. 4)*.* During late ice cover (starting in May), we observed higher contribution of SAFA and less PUFA for *C. scutifer*. This was not observed for *E. graciloides*, where the relative FA composition remained more stable with only a small decrease in PUFA and a slight increase in MUFA and SAFA towards the end of the ice cover. The relative FA contributions of POM decreased in SAFA and a concurrent increase in PUFA and MUFA was observed towards the end of the ice-covered period. The FA content of POM L^−1^ decreased slightly until May and increased during May and June (Fig. S4). Within PUFA, 18:4n-3 was particularly high in *E. graciloides* at the start of ice cover (77.8 ± 3.6 μg mg^−1^ dw; i.e. ~ 17% of total FAME) and rapidly declined with the lowest value found on 04 May 2021 (29.6 ± 5.8 μg mg^−1^ dw), before slightly increasing again in June (37.3 ± 4.4 μg mg^−1^ dw) (Fig. S3, Table S3). There was a large difference between total lipids and total FA for *C. scutifer* (Fig. 4). In January and February, *C. scutifer* had a higher total lipid content that declined thereafter, whereas in *E. graciloides* total lipids followed the same pattern as total FAME. Both total FAME and total lipids declined during ice cover and remained stable from May to June in *E. graciloides*, but they continued to decline in *C. scutifer*.

**Fig. 5:**
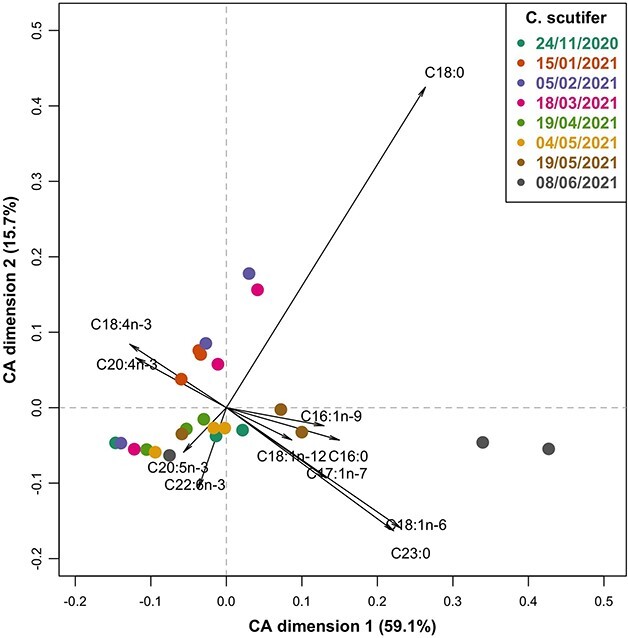
Contribution biplot of CA of the fatty acid profiles of *Cyclops scutifer* (% contribution to total FA content). Individual zooplankton samples are shown as points, with color representing sampling date. FA labels (black) are included when they contribute more to the inertia than expected if all FA were equal, with the exception of C20:5n-3 that was added manually to allow for evaluation of the contribution of this important PUFA.

**Fig. 6:**
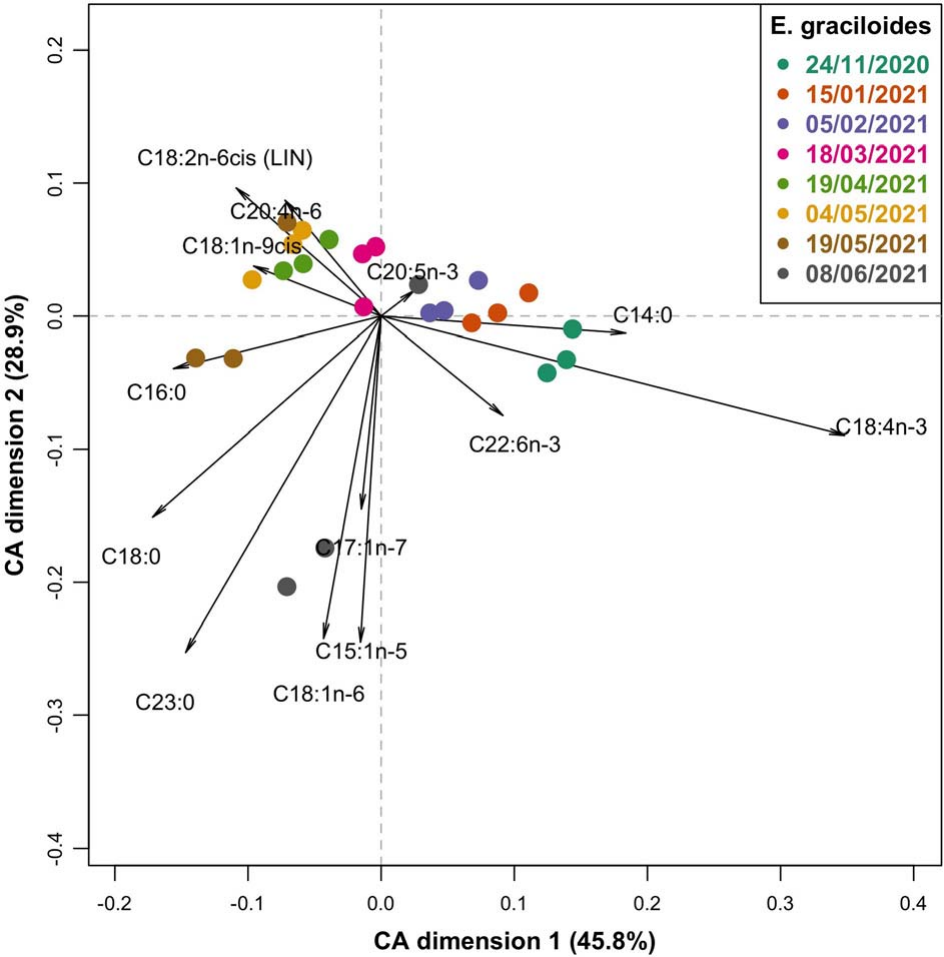
Contribution biplot of CA of the fatty acid profiles of *Euaugaptilus graciloides* (% contribution to total FA content). Formatting of points, colors and labels is the same as for Fig. 5.

The CCA displayed clear interspecific differences in the FA composition between *E. graciloides* and *C. scutifer*, with separation of the two species along the first axis (Fig. S5). Seasonal changes in FA composition were also apparent based on the separation of sampling dates along second axis (Fig. S5). In addition, the results of hierarchical clustering highlights differences in FA composition between POM and zooplankton (Fig. S6). The species separate along axis 1 primarily because of higher contribution of 18:3n-3, 18:1n-9, 18:2n-6 and 18:4n-3 to FA in *E. graciloides* and higher long-chain PUFA (20:4n-3, 22:5n3 and 22:1n-9) in *C. scutifer*. There was a shift for both species towards a higher contribution of SAFA and MUFA (16:1n-9, 17:1n-7, 16:0, 23:0, 18:0 and 18:1n-6) towards the end of the ice-covered period, which is reflected in the second dimension of the CCA (Fig. S5). The first two dimensions (axes) accounted for 83% of the total inertia. Permutation tests of the CCA model (*P* = 0.001) and of the marginal effects of species (*P* = 0.001) and sampling date (*P* = 0.001) on FA composition were significant. Particulate matter and phytoplankton biomass were low throughout the study period. Permutation tests following a CCA on FA composition showed significant marginal effects of sample type (POM *vs.* zooplankton; *P* = 0.001), but not sampling time (*P* = 0.157). The CA biplot highlights a seasonal pattern within the FA composition of *C. scutifer* (Fig. 5). In January, February and March, *C. scutifer* tended to have a higher proportion 18:4n-3 and 20:4n-3 compared to other dates, as well as higher 18:0. In April and May, a relative increase in 20:5n-3 and 22:6n-3 was recorded in *C. scutifer*. The last samples in May and June had higher relative contributions of 18:1n-12, 16:1n-9, 16:0, 17:1n-7, 23:0 and 18:1n-6. Permutation testing following a CCA of the *C. scutifer* FA composition showed a significant effect of sampling date (*P* = 0.006). The FA composition of *E. graciloides* followed a similar seasonal trajectory to that observed in *C. scutifer* (Fig. 6)*.* However, compared to *C. scutifer*, *E. graciloides* displayed a more pronounced seasonal pattern with less variability among triplicate samples starting in November from more PUFA towards more MUFA and SAFA. Permutation testing following a CCA on FA composition of *E. graciloides* showed a significant effect of sampling date (*P* = 0.001).

## DISCUSSION

We studied the importance of storage lipids for zooplankton survival under ice. Our findings emphasize the importance of previously acquired lipids and demonstrate differences in lipid composition and winter usage between two copepod species with different life histories. Algal derived omega-3 PUFA predominantly influence the seasonal variation in lipids of both copepods. There was a large decrease in total lipids during the ice-covered period. Eudiaptomus had noticeably high levels of 18:4n-3.

### Environmental conditions and plankton dynamics

Ice and snow cover drive lake environmental conditions and plankton dynamics, and play a crucial role in controlling primary production (Hampton *et al.*, 2017). During the present study in subarctic Lake Takvatn from 2020 to 2021, the winter snow cover was unusually low. The chl-*a* concentrations were low but comparable with other oligotrophic subarctic lakes just before and after ice-off (Forsström *et al.*, 2005) and with the under-ice conditions in high-latitude lakes (Hampton *et al.*, 2017). Previous studies have also indicated low chl-*a* concentrations in Lake Takvatn, with peak summer concentrations of approximately 1 μg/L (Lyche Solheim *et al.*, 2019). Cold oligotrophic lakes are often dominated by flagellates (crysophytes and dinoflagellates) in the open water season (Trifonova, 1998; Forsström *et al.*, 2005). In Lake Takvatn, dinoflagellates and cyanobacteria were present during winter and were most abundant in April to May directly below the ice, suggesting favorable conditions, e.g. due to higher light intensity and/or convective mixing that could retain cells in the upper water column. Primary production can occur under ice (Jewson *et al.*, 2009; Vehmaa and Salonen, 2009). The current study shows that there is primary production under late ice-cover, which at Lake Takvatn is a period with many hours of daylight and the midnight sun being present from May 18, 2021. Primary production was indicated by an increase in chl-*a* concentrations, an increase in dissolved oxygen and a higher abundance of phytoplankton.

For some phytoplankton, the optimal irradiance (after which growth declines) is as low as 20 μmol m^−2^ s^−1^ (Edwards *et al.*, 2015), which were not uncommon for short periods under ice cover in April in Lake Takvatn. Although several marine studies have reported minimum light thresholds that are much lower than this, including values <10 μmol m^−2^ s^−1^ (Gosselin *et al.*, 1986; Hancke *et al.*, 2018). Many phytoplankton taxa are able to grow at light levels as low as 17 μmol m^−2^ s^−1^ for a 12.5-h daylength (Köhler *et al.*, 2018), which are similar to levels under ice cover that were regularly observed from the end of April onwards during our study. In marine environments the lower threshold for microalgal growth can be as low as 1 μmol m^−2^ s^−1^ (Hoppe, 2022). The low abundance of phytoplankton might in addition to low light availability be the result of a potential grazing effect by zooplankton, which may be particularly pronounced during late ice cover when increased light availability and convective mixing in the surface waters can support enhanced primary production.

In the current study, the winter zooplankton biomass was much lower than previously observed during summer in Lake Takvatn (Dahl-Hansen, 1995), and declined during the ice-covered period due to a combination of lipid depletion and a decrease in abundance. The decreased abundance is consistent with observations from a recent study in a Norwegian subalpine oligotrophic lake (Jensen, 2019). *E. graciloides* reproduced under the ice during March–June which corresponds well with the findings of a previous study in Lake Takvatn (Primicerio and Klemetsen, 1999). There is a latitudinal gradient in the life cycle length of *C. scutifer* (ranging from 0.5 to 3-year cycles, longer at higher latitudes) that is dependent on temperature (Elgmork, 2004). The reproductive timing is dependent on environmental conditions (Vanderploeg *et al.*, 1992) and the availability of phytoplankton (Rivier, 2005), as also previously seen in Lake Takvatn (Abdurhman Kelil, 2007). The life history traits and phenology of these copepods can be constrained by the environment, with a change in climate conditions the copepods possibly shift their reproductive timing similar to that at lower latitudes. The release of nauplii larvae coincided with an increase in chl-*a* possibly in response to increased availability of high-quality phytoplankton. Similar to what has been observed in marine systems where the females took advantage of the ice algal bloom and the offspring of the phytoplankton bloom 2 months later (Søreide *et al.*, 2010).

In contrast to *E. graciloides*, *C. scutifer* was predominantly present in the C-IV to C-V stage, which is consistent with previous observations from Lake Takvatn, where their reproduction has been reported to occur during late June–July (Primicerio, 2000). The zooplankton biomass and community data showed lower zooplankton abundance in the coldest water at <1°C (30-0 m) compared to deeper and warmer water at 2–3°C (30–60 m). This is surprising because staying in colder waters could keep metabolic costs down during overwintering (Koussoroplis *et al.*, 2014). A possible explanation for the movement to deeper and warmer water could be predator avoidance because the zooplankton had brightly colored lipids, combined with low food availability in the upper water column which likely reduces predation in deeper darker waters. The colored lipid droplets in *E. graciloides*, and to a lesser degree in *C. scutifer*, were bright orange and have also been observed for zooplankton under ice in other northern regions, including Russia (Pasternak, 1999) and Canada (Grosbois *et al.*, 2017). Rotifer abundance was lower towards the end compared to the start of ice-cover, possibly due to the low bacterial production in Lake Takvatn, limiting the availability of energy derived through the microbial loop.

### Lipid dynamics in POM

Overall, there was a low availability of FA in POM in the water column during the study period. Contrary to our expectations, bacterial FA in POM were low and relatively stable during ice-cover. Bacterial and terrestrial FA both increased in June coinciding with ice-off and mixing and increased allochthonous inputs via stream inflow, which, in this region, typically occurs during May–June (Poste *et al.*, 2021). Lake Takvatn is oligotrophic with relatively low input of both particulate and dissolved terrestrial material, likely reducing the potential importance of bacterial production and heterotrophy/mixotrophy during winter compared to brown-water lakes that receive substantial input of terrestrial organic matter (Taipale *et al.*, 2018). The FA content of POM (μg L^−1^) in Lake Takvatn was lower than observed in a Canadian boreal lake with a similar ice-cover period but higher nutrient concentrations (Grosbois *et al.*, 2017). The FA composition of POM is affected by environmental conditions, in addition to the community composition. Macronutrient concentrations, i.e. the lake’s trophic status, supposedly play a far greater role in this context than the mere duration of its ice-covered period.

Cryptophytes and chlorophytes are high in 18:3n-6 (alpha-linolenic acid) and 18:4n-3 (Brett *et al.*, 2009; Jónasdóttir, 2019), and cryptophytes are a highly suitable diet for *Eudiaptomus* with regard to reproduction (Von Elert and Stampfl, 2000). In early May, there was a marked increase in PUFA in the POM that coincided with increased light availability, phytoplankton abundance and chl-*a* concentration. The lack of apparent decrease of nutrients in May suggests that phytoplankton are likely not nutrient limited at this point. The main phytoplankton taxa observed during late ice cover were cryptophytes, cyanobacteria, chlorophytes and larger diatoms (Fig. S2). In May–June, the concentrations of POM-associated FA in the water column increased, especially for 18:4n-3, which is found in cryptophytes; 20:6n-3 [Docosahexaenoic acid (DHA)], which is among the dominating PUFA in dinoflagellates and chrysophytes and 20:5n-3, which usually is the major PUFA in diatoms (Taipale *et al.*, 2013). Active grazing is reflected in the FA composition of *E. graciloides*, which increased in PUFA, especially 18:4n-3 from late May to June. In contrast, *C. scutifer* continued declining in PUFA, indicating that the increased availability of phytoplankton did not result in net accumulation of PUFA in this species.

### Lipid dynamics in zooplankton

There was no evidence of significant retention of terrestrial FA by zooplankton in terms of lipid trophic markers. However, our study did not include analysis of longer chained SAFA (e.g. C26, C28), which means that we are likely to have underestimated the terrestrial signal present, both in POM and in zooplankton. The present study indicated that zooplankton were at least partly feeding during the 5-month ice-cover period in Lake Takvatn. This could be seen by the increase in total lipids until February for *C. scutifer* and in June for *E. graciloides*. *C. scutifer* has a raptorial feeding strategy in the later stages (from C-IV onwards) and might feed on microzooplankton (rotifers and nauplii) after the autumn bloom and thus store lipids for a longer period. Rotifers could be an important food source for *C. scutifer* under ice cover because rotifers can contain PUFA depending on their diet (Nichols *et al.*, 1996; Desvilettes *et al.*, 1997; Wacker and Weithoff, 2009). However, *C. scutifer* might also rely more on algal derived FA to store up on lipids at the start of ice cover, this we cannot distinguish with our data set. Our FA analysis did not show what the species were feeding on; however, the expectation was to see a higher bacterial signal due to the possible reliance on rotifers as a food source. In a Canadian lake, autumn and early winter were found to be essential periods for the accumulation of lipids in zooplankton (Mariash *et al.*, 2016; Grosbois *et al.*, 2017). Other studies from Russia and Finland (Hiltunen *et al.*, 2014, 2015; Perga *et al.*, 2020) also show the importance of previously accumulated FA for actively overwintering zooplankton. This is in line with our findings in Lake Takvatn, where *C. scutifer* reached the highest total lipid content in February (up to 73% of dry weight) when the lake had already been ice-covered for nearly 3 months. The highest total lipid content of *E. graciloides* (60%) was measured in November. Grosbois *et al.* (2017) report up to 76% total lipids in copepods under ice cover in January in Lake Simoncouche, suggesting that accumulated lipids play an important role for survival under lake ice cover. In Lake Simoncouche, *C. scutifer* had up to 50% total lipid content of their biomass in January compared to only 21% in May (Grosbois *et al.*, 2017). By June in Lake Takvatn, total lipids had decreased to 33% for *C. scutifer* and 38% for *E. graciloides*, indicating the high importance of previously acquired lipids in moderating the effects of large lipid losses during the long ice-cover period. The importance of previously acquired lipids is further illustrated by the high 18:4n-3 content used during reproduction in *E. graciloides*, similar to a study in Finland reporting high 18:4n-3 in *Eudiaptomus* (Hiltunen *et al.*, 2015)*.* Lake Takvatn is located much farther north than Lake Simoncouche, which could result in higher light availability during late winter if the snow and ice-cover conditions allow. The unusual lack of snow cover on Lake Takvatn during most of this study period may have contributed to a higher food availability, thereby facilitating total lipids of >33%. It is likely that the lipid content at the end of winter can be highly variable depending on environmental and ecophysiological conditions as well as food availability throughout the ice-cover period. Data from 2020 and 2021 for the total lipid content of bulk zooplankton (*E. graciloides* and *C. scutifer* combined) indicate that total lipid content at the end of the ice-covered period in Lake Takvatn in early June was much lower in 2020 (13%) compared to 2021 (27%) (unpubl. Data; Kers *et al.*). Snow cover on top of the lake ice was considerably higher in 2019–2020, likely leading to a delayed start of pelagic primary production at the end of the ice-cover period in that year. However, a long-term (and multi-lake) study would be necessary to shed light on the intricacies of zooplankton lipid content and environmental conditions during ice-cover.

Towards the end of ice cover, the total lipid content in *E. graciloides* remained stable, indicating that during that time lipid metabolism was on par with dietary lipid uptake and loss, whereas the lipid content of *C. scutifer* kept declining. *E. graciloides* possibly fed on a resource that *C. scutifer* cannot utilize since calanoid copepods are filter feeders and can likely utilize the phytoplankton produced under late ice-covered period more efficiently than *C. scutifer* (Ger *et al.*, 2011). In addition, spatial (vertical) separation between *C. scutifer* and its food source might have occurred, yet with our data we are not able to evaluate such separation.

At the start of ice-cover, both copepods were characterized by high lipid contents and high PUFA levels. MUFA and SAFA are major groups for storage lipids, whereas long-chain polyunsaturated fatty acids (LC-PUFA) are mainly incorporated into structural lipids (Arts *et al.*, 2009). Storage of LC-PUFA might be of importance in *E. graciloides* for reproduction under late ice cover as it plays an important role in reproduction for other zooplankton (Schlotz *et al.*, 2012). In contrast, our results showed a higher contribution of LC-PUFA in *C. scutifer*. The higher trophic position of *C. scutifer* may be of importance here, possibly causing an increase in LC-PUFA (Persson and Vrede, 2006). As *C. scutifer* is not in the final life stage during the ice-covered period, it is likely that instead of storing LC-PUFA for reproduction it uses the spring bloom to invest in growth to reach the adult stage and consequently reproduce in summer.

The high PUFA content in early winter was followed by a quick decrease in *E. graciloides* and corresponded with an increase in egg-carrying females and egg release. The very high values of 18:4n-3 up to 17% of total FAME and 78 μg mg^−1^ in *E. graciloides* decreased by >50% during this reproductive period and increased only slightly again towards the end of ice cover when pelagic primary production increased. The 18:4n-3 contents of *E. graciloides* were about 4 times higher compared to those previously reported for the adult calanoid copepod *Leptodiaptomus minutus* (19 μg mg^−1^) at the start of ice-cover (Grosbois *et al.*, 2017). Similar contents of 18:4n-3 in *Eudiaptomus* have been observed in other subarctic lakes (Hiltunen *et al.*, 2016), whereas *Eudiaptomus* in boreal lakes had in contrast much lower values of 18:4n-3 (Hiltunen *et al.*, 2016), which might be related to lipid accumulation before ice cover or differences in food availability in the subarctic systems. It is possible that 18:4n-3 is used to bioconvert to omega-3 LC-PUFA because 18:4n-3 is a precursor for the LC-PUFA 20:5n-3 and 20:6n-3 (DHA) (Guil-Guerrero, 2007; Peltomaa *et al.*, 2017). Cryptophytes contain a relatively large amount of 18:4n-3 (Taipale *et al.*, 2013), and cryptophytes were abundant under ice cover (Fig. 2). It is likely that *Eudiaptomus* highly invests PUFA (possibly mainly SDA) and MUFA into egg production, as seen in other copepods (Schneider *et al.*, 2017) and *Daphnia* (Arts *et al.*, 2009). *E. graciloides* decreased most in 18:4n-3 from March to May, coinciding with females releasing eggs and suggesting the use of the capital breeding strategy (Schneider *et al.*, 2017).

### Future scenarios

There are many possible changes to the ice-covered season in a warming climate. A prolonged ice-free period and increase in primary production is expected with an increase in temperature (Karlsson *et al.*, 2005). In addition, more frequent rain events during the ice-covered period will also increase transmittance of snow and ice, and lead to increased light availability in the water column underneath (Hébert *et al.*, 2021). It has been suggested that the biomass production of phytoplankton is related to the length of the ice-free season, more so than weather conditions and thermal stability (Forsström *et al.*, 2005), but total annual production will also be limited by the nutrients available. Some phytoplankton taxa (especially in shallow lakes) might benefit from a milder and shorter winter with a better-mixed water column, while this could be disadvantageous to mixotrophic plankton, which in contrast would benefit from a severe winter that favors motile taxa and limits phototrophic taxa (Özkundakci *et al.*, 2016). Besides an increase in primary production there might be negative effects for actively overwintering zooplankton. A decrease in light availability due to white ice (Weyhenmeyer *et al.*, 2022) could result in less primary production under ice cover. In addition, trophic mismatch of food availability and consumer reproduction might occur due to earlier offset of spring phytoplankton blooms, and this effect might be especially strong in lakes with ice cover (Peeters *et al.*, 2007). For *E. graciloides*, a mismatch between the release of nauplii and the spring phytoplankton bloom can be detrimental to nauplii survival. For *C. scutifer*, however, this might have a positive impact by having earlier access to food. There could be a decrease in thermal specialist zooplankton (such as polar or tropical specialists) due to climate change (Dam and Baumann, 2017). Climate change can lead to changes in phyto- and zooplankton community structure, where cold-water adapted zooplankton, such as *C. scutifer* and *E. graciloides*, may coexist with warm water adapted species possibly increasing competition and/or new stratification regimes (McMeans *et al.*, 2020). This is also the case for higher trophic levels such as fish where some winter specialists (e.g. salmonids) actively feed during the winter (Shuter *et al.*, 2012).

Overall, it is important to understand the current under-ice conditions and to acknowledge how the community composition and lipid composition might change in the future. Zooplankton are key consumers of the pelagic food web and an important source of dietary energy to consumers at higher trophic levels (including fish and humans), and changes in abundance, lipid content and composition can have a broad range of consequences for lake ecosystems.

## CONCLUSION

This lake study indicates active feeding and the importance of retained lipids for successful survival of actively overwintering freshwater copepods. At low water temperatures, PUFA play a vital role and were the most abundant FA group in both copepods during the study period. In addition, a more dynamic and pronounced drawdown of dietary PUFA (in particular 18:4n3) was found in reproducing *E. graciloides*. The importance of total lipids and high-quality algal-derived PUFA for survival is also in agreement with the findings of other studies (Grosbois *et al.*, 2017; Hébert *et al.*, 2021). The two actively overwintering copepods in Lake Takvatn have different life history strategies, where the release of nauplii of *E. graciloides* coincides with increased under-ice primary production under late ice-cover, whereas *C. scutifer* does not reproduce under ice cover.

Contrary to the hypothesis, the reproducing *E. graciloides* did not have a larger decrease in total lipids compared to *C. scutifer* although it exhibited a large decrease in total FAME. The FA composition differed between the species and showed monthly changes throughout the study period. The changes in lipid contents and composition suggested that *C. scutifer* may have been feeding actively during the early ice-cover period, while *E. graciloides* was feeding actively during the late ice-cover period.

## Supplementary Material

Supplementary_material_fbae018
