# Correction to: Under ice plankton and lipid dynamics in a subarctic lake

**DOI:** 10.1093/plankt/fbae037

**Published:** 2024-06-28

This is a correction to: Erwin Kers, Eva Leu, Per-Arne Amundsen, Raul Primicerio, Martin Kainz, Amanda E Poste, Under ice plankton and lipid dynamics in a subarctic lake, *Journal of Plankton Research*, Volume 46, Issue 3, May/June 2024, Pages 323–337, https://doi.org/10.1093/plankt/fbae018

In the originally published version of this article, there was an error in the legend of Figure 3, whereby *Eudiaptomus graciloides* was misspelled as *Euaugaptilus graciloides*.

This has been corrected.

