# Supplementary material for: Under ice plankton and lipid dynamics in a subarctic lake
Source: J Plankton Res. 2024 May 3;46(3):323–37. doi: 10.1093/plankt/fbae018 (PMC11142452; doi:10.1093/plankt/fbae018)
Supplement: Supplementary_material_fbae018 [file supplementary_material_fbae018.docx]

# Supplementary material

## Map of study location


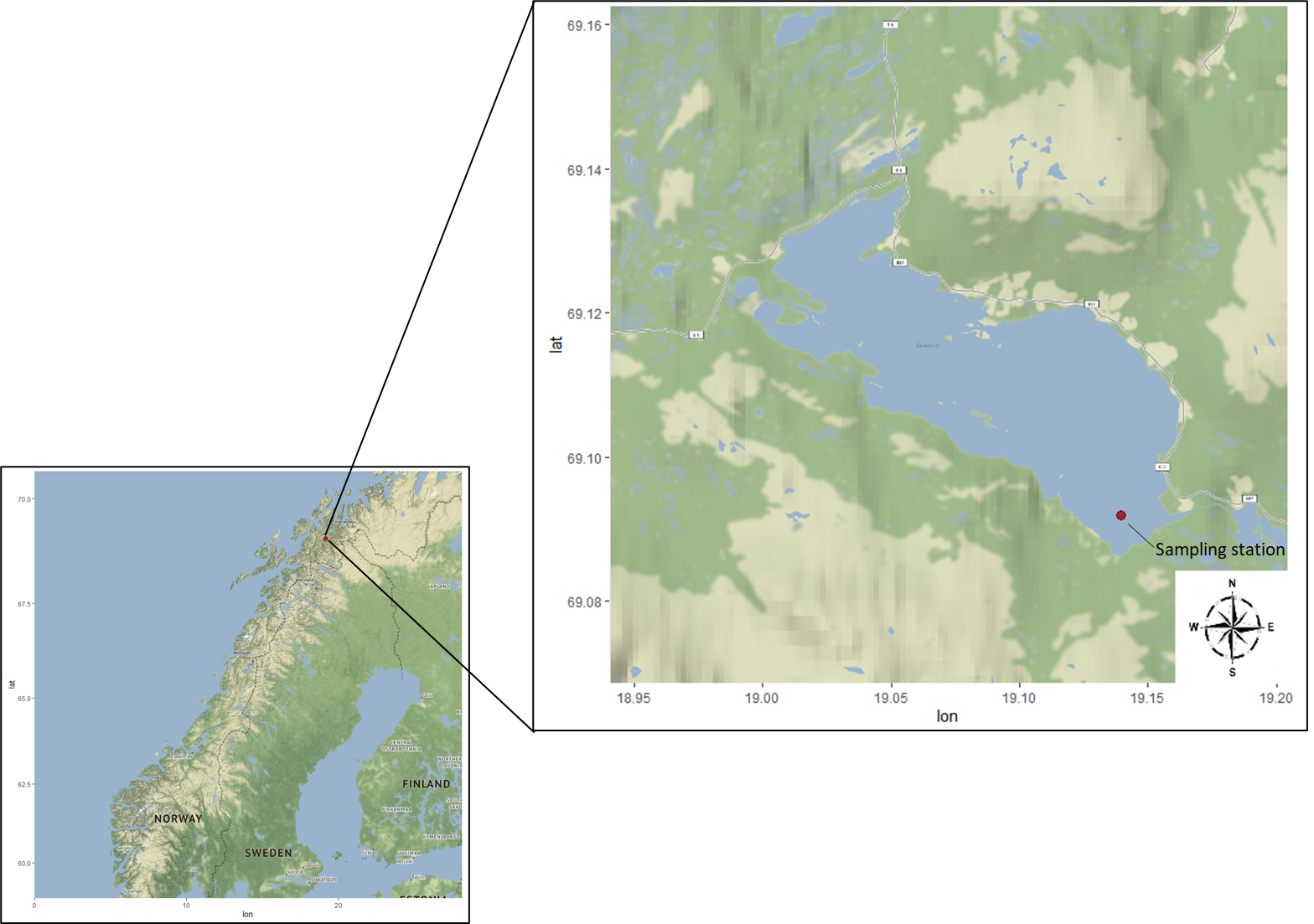


Figure S1. Map of Takvatn, red dot indicates sampling location (Lat: 69.09201, Lon: 19.13962)

## Physical conditions

Table S1. Observed physical conditions for each sampling date.

| **Date** | **Secchi depth (m)** | **Snow depth (cm)** | **Ice thickness (cm)** | **Conditions** |
| --- | --- | --- | --- | --- |
| 27-10-2020 | 11.5 | - | - | Open water |
| 24-11-2020 | 10.5 | - | - | Open water |
| 15-01-2021 | 14 | 0-0,5 | 42.5 | Clear ice |
| 05-02-2021 | 11 | 4.5 | 62.5 | Dry snow |
| 18-03-2021 | 10 | 21 | 73 | Dry snow |
| 19-04-2021 | 11.5 | 8 | 65 | Slush, small hard layer on top |
| 04-05-2021 | 10.5 | 4.2 | 69 | Thick frozen slush (20cm) |
| 19-05-2021 | 18 | 0 | 56 | Dry ice, sharp point at the top. |
| 08-06-2021 | 15 | - | - | Open water at the sampling site. |

Table S2. Nutrient data in Takvatn of the vertical 0-10 meters of the water column and at 58-meter depth, 2 meters above the bottom.

| **Date** | **Sample depth**  **(m)** | **Phosphate**  **(µg L-1)** | **Nitrate+Nitrite**  **(µg L-1)** | **Silicate**  **(µg L-1)** |
| --- | --- | --- | --- | --- |
| 27.10.2020 | 0-10 | 2 | 47 | 770 |
| 24.11.2020 | 0-10 | 4 | 50 | 750 |
| 15.01.2021 | 0-10 | 2 | 55 | 810 |
| 05.02.2021 | 0-10 | 2 | 57 | 810 |
| 18.03.2021 | 0-10 | 2 | 60 | 800 |
| 19.04.2021 | 0-10 | 2 | 58 | 810 |
| 04.05.2021 | 0-10 | 1 | 59 | 820 |
| 19.05.2021 | 0-10 | 2 | 62 | 820 |
| 08.06.2021 | 0-10 | 1 | 58 | 830 |
| 27.10.2020 | 58 | 4 | 48 | 760 |
| 24.11.2020 | 58 | 2 | 51 | 760 |
| 15.01.2021 | 58 | 3 | 56 | 820 |
| 05.02.2021 | 58 | 2 | 63 | 860 |
| 18.03.2021 | 58 | 2 | 72 | 870 |
| 19.04.2021 | 58 | 2 | 82 | 930 |
| 04.05.2021 | 58 | 1 | 70 | 830 |
| 19.05.2021 | 58 | 2 | 78 | 890 |
| 08.06.2021 | 58 | 1 | 63 | 870 |


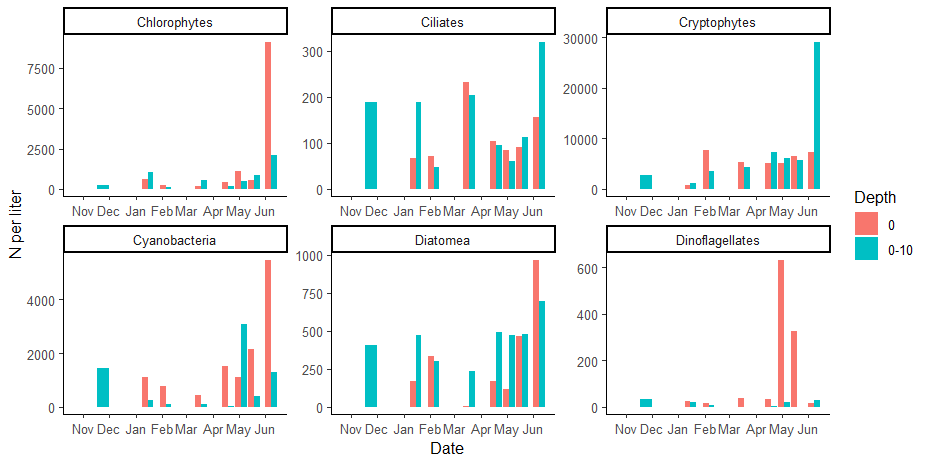


Figure S2. Protist species composition in numbers per liter, samples were taken at 0 meters: just below ice and integrated 0-10 meters.

## Fatty acids


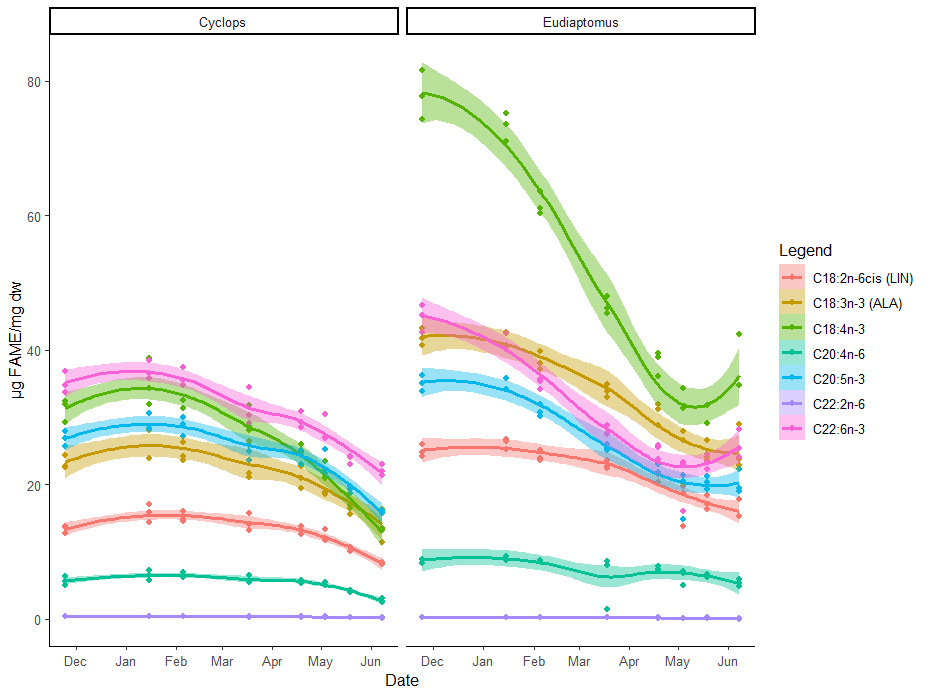


Figure S3. Y-axis shows the fatty acids in µg/mg dry weight for zooplankton. The confidence interval (95%) is visualized by the light-coloured area around the lines.


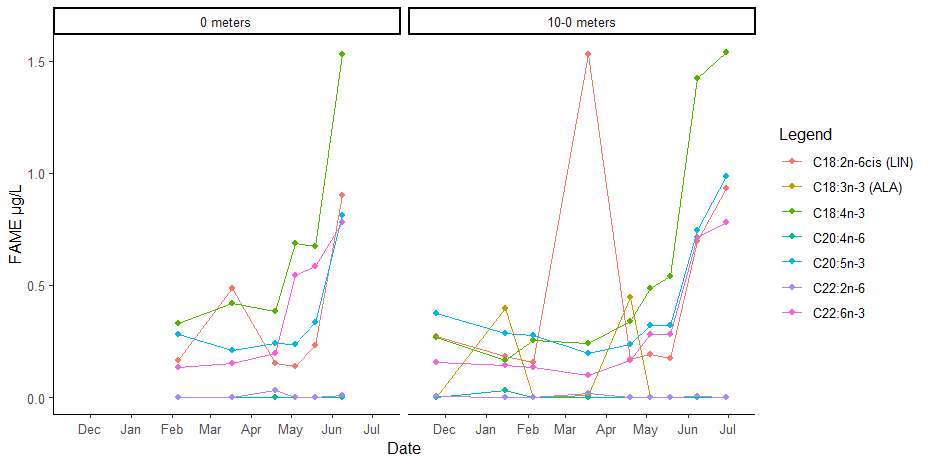


Figure S4. Y-axis shows the fatty acids in µg L^-1^ of water filtered. Note that measurements were only taken at the dots and the lines are a generalization to visualize the pattern over time.


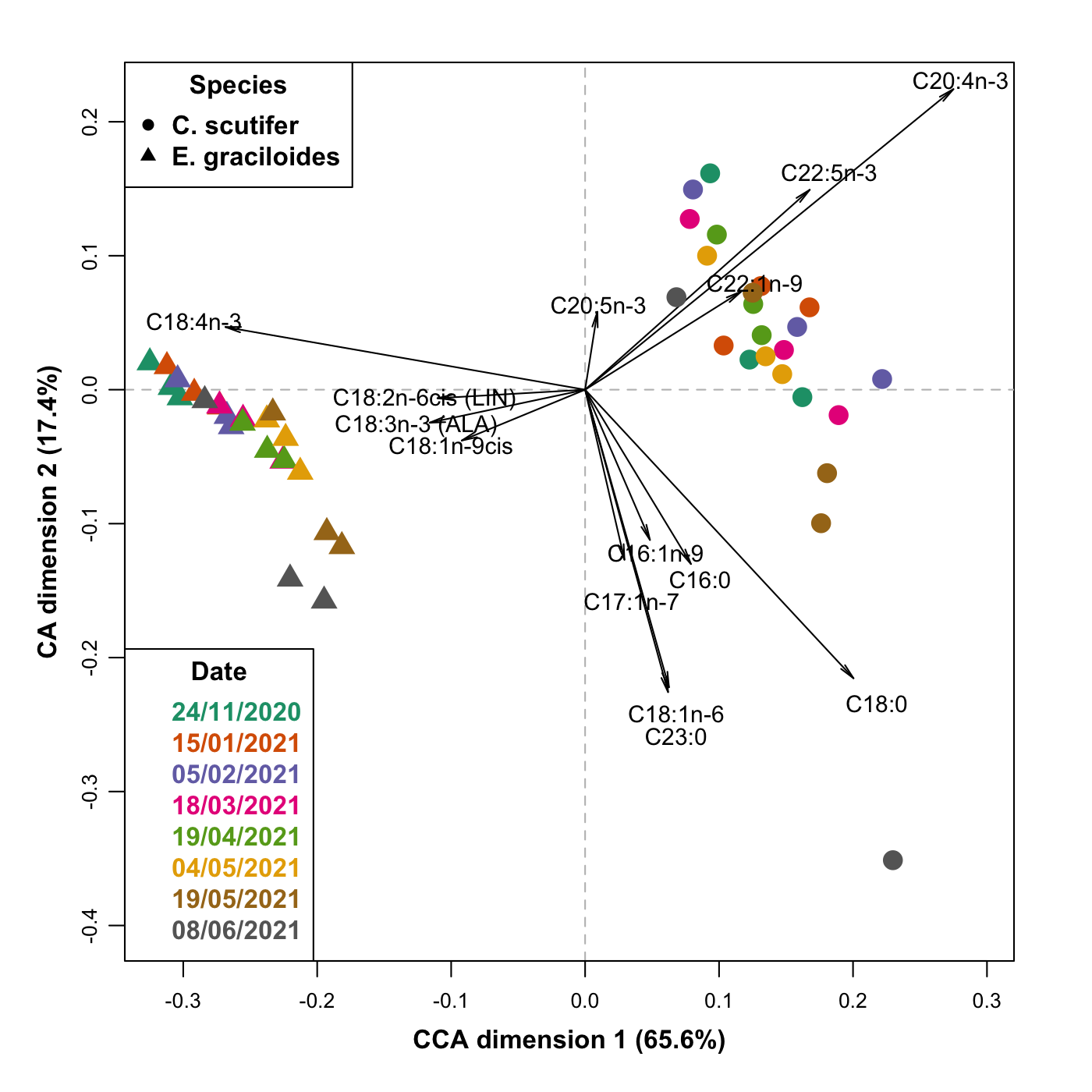


Figure S5. Canonical correspondence analysis of the fatty acid profiles of E. graciloides and C. scutifer, with sampling date indicated by colour and species indicated by shape. The first dimension (axis) contributes to 65.6% and the second dimension to 17.4% of the total inertia. FA labels are shown when they contribute more to the inertia than expected if all FA were equal, except for C20:5n-3 which was added manually was added manually to allow for evaluation of the contribution of this important PUFA.


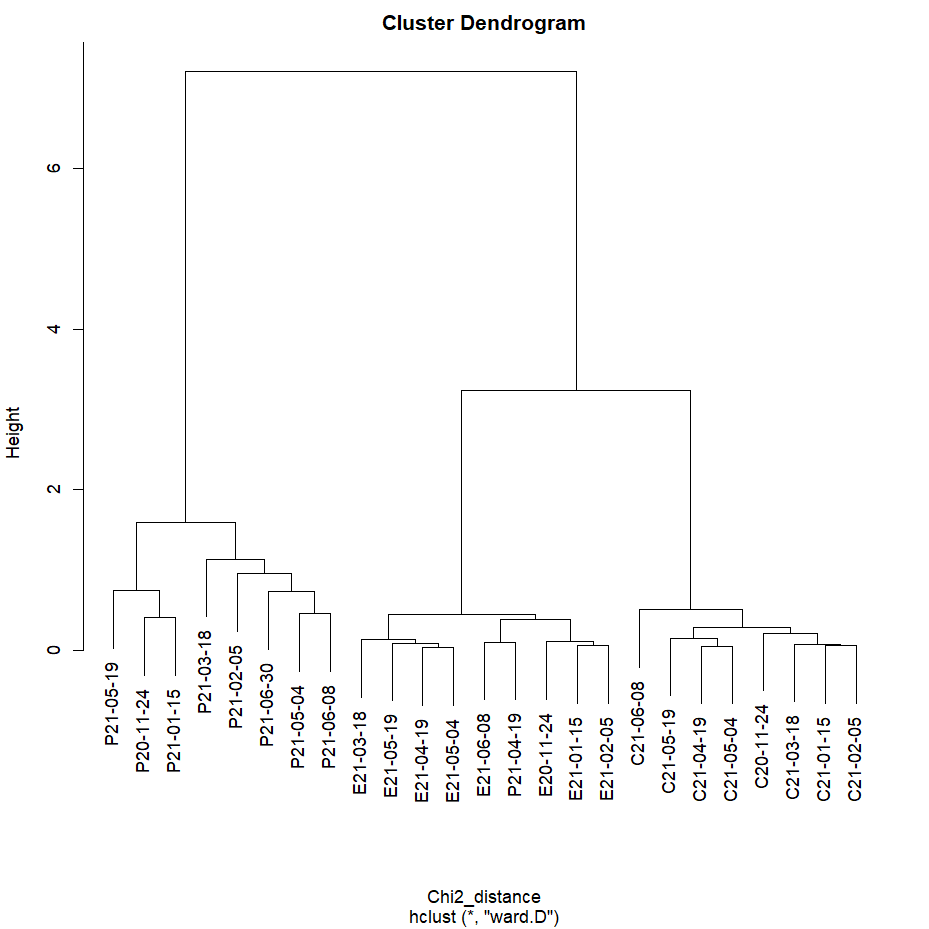


Figure S6. Cluster dendrogram visualizing the difference between species and dates from different samples. Mean values per date were used. E: E. graciloides, C: C. scutifer, P: Particulate organic matter. Mean values per date were used from the compositional FA data.

## Fatty acid composition data

Table S3. Fatty acid concentrations in % of total FA, except for total lipids which is depicted in µg mg DW^-1^, mean ± standard deviation, n= number of subsamples. n.d.= not detected.

| Date | 24-11-2020 | 24-11-2020 | 24-11-2020 | 15-1-2021 | 15-1-2021 | 15-1-2021 |
| --- | --- | --- | --- | --- | --- | --- |
| Species | C. scutifer | E. graciloides | POM | C. scutifer | E. graciloides | POM |
| Sub samples | 3 | 3 | 1 | 3 | 3 | 1 |
| Total_lipids | 474.96±23.32 | 584.27±11.49 | NaN±NaN | 666.6±32.04 | 585.54±1.08 | NaN±NaN |
| C14:0 | 10.14±0.33 | 12.85±0.16 | 2.22 | 9.53±0.33 | 12.11±0.26 | 3.06 |
| iso-15:0 | 1.48±0.09 | 1.24±0.01 | 0.81 | 1.36±0.03 | 1.28±0.02 | 0.13 |
| anteiso-15:0 | 0.78±0.02 | 0.78±0.01 | 0.42 | 0.78±0.02 | 0.79±0.01 | 0.36 |
| C14:1n-5 | 0.09±0.01 | 0.08±0.02 | 0.08 | 0.09±0.01 | 0.07±0.01 | 0.06 |
| C15:0 | 0.83±0.02 | 0.6±0.01 | 0.47 | 0.79±0.01 | 0.63±0 | 0.44 |
| iso-16:0 | 0.78±0.24 | 0.4±0.08 | 0.17 | 0.64±0.05 | 0.51±0 | 0.25 |
| C15:1n-5 | 0.11±0.09 | 0.03±0.01 | n.d. | 0.02±0.01 | 0±0.01 | n.d. |
| C16:0 | 15.49±1.61 | 13.16±0.44 | 25.74 | 15.23±1.09 | 13.35±0.17 | 29.21 |
| iso-17:0 | 0.32±0.02 | 0.3±0 | 0.54 | 0.33±0.01 | 0.31±0 | 0.18 |
| C16:1n-9 | 0.44±0.08 | 0.37±0.01 | 0.53 | 0.6±0.1 | 0.4±0.02 | 0.83 |
| C16:1n-7 | 4.04±0.14 | 4.39±0.03 | 7.22 | 4.1±0.11 | 4.65±0.03 | 3.05 |
| C17:0 | 0.34±0.02 | 0.32±0.02 | 0.36 | 0.41±0.04 | 0.35±0.02 | 0.36 |
| 9,10D16 | n.d. | 0.05±0.09 | n.d. | n.d. | n.d. | n.d. |
| C17:1n-7 | 0.13±0.09 | 0.05±0 | n.d. | 0.05±0.02 | 0.03±0 | n.d. |
| C18:0 | 5±1.2 | 2.73±0.32 | 43.93 | 7.05±0.82 | 3.32±0.7 | 43 |
| C18:1n-9trans | 0.13±0.02 | 0.11±0.01 | 0.11 | 0.18±0.01 | 0.13±0.02 | n.d. |
| C18:1n-12 | 0.03±0.05 | 0.02±0.03 | 0.28 | n.d. | 0.03±0 | 0.21 |
| C18:1n-9cis | 3.48±0.2 | 4.84±0.17 | 2.27 | 3.74±0.15 | 5±0.01 | 3.63 |
| C18:1n-7 | 1.71±0.27 | 1.47±0.04 | 0.94 | 2.14±0.3 | 1.61±0.03 | 2.29 |
| C18:1n-6 | 0.49±0.35 | 0.14±0.07 | 0.74 | 0.1±0.01 | 0.05±0 | n.d. |
| C19:0 | 0.59±0.15 | 0.36±0.03 | 0.31 | 0.45±0.14 | 0.27±0.01 | 1.07 |
| C18:2n-6trans | n.d. | 0±0.01 | n.d. | n.d. | 0.01±0.01 | 0.05 |
| 9,10D18 | 0.09±0.01 | 0.13±0 | n.d. | 0.08±0 | 0.12±0 | 0.09 |
| C18:2n-6cis (LIN) | 4.18±0.21 | 5.56±0.07 | 1.44 | 4.32±0.14 | 5.85±0.07 | 1.24 |
| C20:0 | 0.43±0.02 | 0.39±0.01 | 0.59 | 0.55±0.02 | 0.43±0.02 | 0.51 |
| C18:3n-6 | 0.29±0.02 | 0.63±0.02 | 0.17 | 0.36±0.1 | 0.64±0.01 | 0.08 |
| C20:1n-9 | n.d. | n.d. | 2.92 | n.d. | n.d. | n.d. |
| C18:3n-3 (ALA) | 7.26±0.2 | 9.32±0.03 | n.d. | 7.31±0.27 | 9.39±0.09 | 3.23 |
| C21:0 | 0.03±0.05 | 0.01±0.02 | 0.03 | 0.12±0.09 | 0.03±0 | 0.28 |
| C18:4n-3 | 9.77±0.64 | 17.31±0.3 | 1.4 | 9.6±0.5 | 16.45±0.29 | 2.46 |
| C20:2n-6 | 0.55±0.04 | 0.44±0 | 0.11 | 0.57±0.01 | 0.47±0.01 | 0.08 |
| C22:0 | 0.21±0.01 | 0.2±0.01 | 0.41 | 0.26±0.03 | 0.23±0.01 | 0.27 |
| C20:3n-6 | 0.2±0.01 | 0.1±0 | 0.69 | 0.2±0.02 | 0.11±0 | n.d. |
| C22:1n-9 | 0.72±0.04 | 0.06±0.01 | 0.03 | 0.7±0.06 | 0.06±0 | n.d. |
| C20:3n-3 | 0.21±0.03 | 0.11±0.01 | 1.81 | 0.22±0.02 | 0.13±0 | 0.18 |
| C20:4n-6 | 1.77±0.25 | 1.94±0.1 | 0 | 1.78±0.19 | 2.04±0.02 | n.d. |
| C23:0 | 0.02±0.03 | 0.02±0.03 | 0.07 | 0.05±0.02 | 0.03±0.01 | 0.03 |
| C20:4n-3 | 4.93±0.36 | 0.51±0.03 | 0.12 | 4.66±0.45 | 0.55±0.05 | 0.22 |
| C22:2n-6 | 0.1±0 | 0.04±0 | 0.02 | 0.1±0.01 | 0.04±0.01 | n.d. |
| C24:0 | 0.11±0.01 | 0.06±0.01 | 0.38 | 0.13±0.01 | 0.07±0.01 | 0.26 |
| C20:5n-3 | 8.4±0.49 | 7.79±0.04 | 1.82 | 8±0.34 | 7.91±0.11 | 1.58 |
| C24:1n-9 | 0.69±0.04 | 0.48±0.02 | n.d. | 0.72±0.02 | 0.54±0.01 | 0.1 |
| C22:3n-3 | 0.28±0.04 | 0.14±0.01 | n.d. | 0.3±0.03 | 0.15±0.01 | n.d. |
| C22:4n-6 | n.d. | 0±0.01 | n.d. | n.d. | n.d. | n.d. |
| C22:5n-3 | 2.36±0.15 | 0.48±0.01 | 0.09 | 2.23±0.17 | 0.47±0.01 | 0.12 |
| C22:6n-3 | 11.01±0.72 | 9.96±0.15 | 0.76 | 10.15±0.35 | 9.4±0.14 | 1.11 |

| Date | 5-2-2021 | 5-2-2021 | 5-2-2021 | 18-3-2021 | 18-3-2021 | 18-3-2021 | 19-4-2021 | 19-4-2021 | 19-4-2021 |
| --- | --- | --- | --- | --- | --- | --- | --- | --- | --- |
| Species | C. scutifer | E. graciloides | POM | C. scutifer | E. graciloides | POM | Cyclops | E. graciloides | POM |
| Sub samples | 3 | 3 | 2 | 3 | 3 | 2 | 3 | 3 | 2 |
| Total_lipids | 610.18±24.49 | 561.94±20.22 | NaN±NaN | 571.92±24.38 | 467.77±8 | NaN±NaN | 442.46±34.03 | 420.4±32.79 | NaN±NaN |
| C14:0 | 10.44±0.74 | 12.3±0.12 | 4.3±0.75 | 10.6±0.56 | 11.76±0.35 | 3.52±0.03 | 10.15±0.31 | 11.05±0.12 | 6.73±5.61 |
| iso-15:0 | 1.47±0.1 | 1.37±0.04 | 1.93±0.72 | 1.51±0.09 | 1.48±0.04 | 1.86±0.3 | 1.58±0.02 | 1.52±0.03 | 1.42±0.14 |
| anteiso-15:0 | 0.86±0.05 | 0.82±0.01 | 0.79±0.13 | 0.88±0.04 | 0.89±0.01 | 0.67±0.07 | 0.88±0.03 | 0.91±0.01 | 0.63±0.19 |
| C14:1n-5 | 0.1±0 | 0.07±0.01 | 0.07±0.09 | 0.11±0.01 | 0.08±0 | 0.06±0.09 | 0.09±0.02 | 0.08±0 | 0.11±0.07 |
| C15:0 | 0.84±0.04 | 0.64±0 | 1.22±0.16 | 0.89±0.02 | 0.66±0 | 1.27±0.01 | 0.91±0.02 | 0.68±0.02 | 0.48±0.13 |
| iso-16:0 | 0.63±0.03 | 0.53±0.01 | 0.22±0.02 | 0.83±0.09 | 0.62±0.04 | 0.39±0.09 | 0.81±0.1 | 0.64±0.02 | 0.31±0.16 |
| C15:1n-5 | 0.01±0.02 | n.d. | n.d. | 0.01±0.02 | n.d. | n.d. | 0.02±0.01 | 0.01±0 | 0.09±0.12 |
| C16:0 | 14.94±1.11 | 13.76±0.21 | 25.78±0.89 | 15.51±0.92 | 14.52±0.51 | 23.34±3.97 | 15.54±0.37 | 15.07±0.47 | 17.96±3.05 |
| iso-17:0 | 0.34±0.04 | 0.31±0.02 | 1.57±1.04 | 0.35±0.03 | 0.37±0 | 1.03±0.01 | 0.38±0.01 | 0.38±0 | 0.67±0.6 |
| C16:1n-9 | 0.55±0.09 | 0.39±0.02 | 1.82±0.39 | 0.55±0.08 | 0.4±0.04 | 1.43±0.08 | 0.59±0.07 | 0.45±0.04 | 0.54±0.23 |
| C16:1n-7 | 4.28±0.32 | 4.81±0.06 | 8.39±1.76 | 4.45±0.29 | 5.12±0.05 | 4.1±0.98 | 4.76±0.16 | 5.2±0.06 | 4.39±0.38 |
| C17:0 | 0.42±0.04 | 0.36±0.02 | 0.57±0.05 | 0.42±0.03 | 0.41±0.01 | 0.67±0.03 | 0.44±0 | 0.44±0 | 0.35±0.08 |
| 9,10D16 | n.d. | 0.01±0.02 | n.d. | n.d. | n.d. | n.d. | n.d. | n.d. | n.d. |
| C17:1n-7 | 0.03±0.02 | 0.03±0 | n.d. | 0.04±0.03 | 0.03±0.01 | n.d. | 0.04±0 | 0.03±0 | 0.08±0.11 |
| C18:0 | 7.31±3.3 | 3.64±0.82 | 24.27±3 | 7.05±3.06 | 3.82±1 | 24.26±9.96 | 4.97±0.86 | 3.68±0.56 | 25.66±28.86 |
| C18:1n-9trans | 0.17±0.04 | 0.14±0.02 | 0.11±0.16 | 0.17±0.01 | 0.16±0.01 | 0.8±0.68 | 0.18±0.01 | 0.18±0.01 | 0.16±0.05 |
| C18:1n-12 | n.d. | 0.01±0.02 | 0.58±0.37 | 0.03±0.05 | 0.02±0.02 | 0.41±0.57 | 0.02±0.02 | 0.03±0.03 | 0.18±0.25 |
| C18:1n-9cis | 3.57±0.31 | 5.19±0.18 | 3.34±0.76 | 3.79±0.23 | 5.69±0.12 | 11.07±5.73 | 4.03±0.1 | 5.77±0.13 | 4.37±1.97 |
| C18:1n-7 | 1.91±0.06 | 1.67±0.03 | 1.34±0.25 | 1.92±0.12 | 1.82±0.06 | 1.49±0.33 | 2.06±0.03 | 1.93±0 | 0.78±1.09 |
| C18:1n-6 | 0.09±0.01 | 0.05±0 | 0.66±0.93 | 0.1±0.01 | 0.05±0 | n.d. | 0.11±0.01 | 0.06±0.01 | 0.21±0.29 |
| C19:0 | 0.42±0.03 | 0.26±0 | 0.73±0.22 | 0.3±0.19 | 0.22±0.01 | 0.7±0.12 | 0.44±0.04 | 0.24±0.02 | 0.59±0.07 |
| C18:2n-6trans | 0.01±0.02 | 0.01±0.01 | n.d. | 0.01±0.01 | 0±0.01 | 0.18±0.25 | 0.02±0.01 | 0±0.01 | n.d. |
| 9,10D18 | 0.07±0.01 | 0.11±0 | n.d. | 0.07±0.01 | 0.1±0 | 0±0 | 0.07±0 | 0.09±0 | 0.09±0.12 |
| C18:2n-6cis (LIN) | 4.22±0.3 | 6.04±0.17 | 1.78±0.26 | 4.41±0.26 | 6.68±0.06 | 8.72±5.91 | 4.53±0.12 | 6.9±0.1 | 3.77±3.37 |
| C20:0 | 0.56±0.09 | 0.44±0.01 | 0.67±0.1 | 0.55±0.08 | 0.46±0.02 | 0.9±0.05 | 0.5±0.01 | 0.42±0.01 | 0.42±0.14 |
| C18:3n-6 | 0.28±0.03 | 0.64±0.01 | 0.22±0.01 | 0.28±0.01 | 0.65±0.01 | 0.07±0.09 | 0.29±0.01 | 0.63±0.01 | 0.32±0.27 |
| C20:1n-9 | n.d. | n.d. | 2.77±0.2 | n.d. | n.d. | 2.28±0.19 | n.d. | n.d. | 1.61±2.27 |
| C18:3n-3 (ALA) | 6.91±0.65 | 9.55±0.13 | n.d. | 7.07±0.54 | 9.91±0.14 | 0.03±0.04 | 7.23±0.04 | 10.02±0.22 | 4.71±6.66 |
| C21:0 | 0.06±0.01 | 0.03±0.01 | n.d. | 0.07±0 | 0.03±0 | 0.07±0.1 | 0.09±0.01 | 0.04±0 | n.d. |
| C18:4n-3 | 9.18±0.58 | 15.4±0.33 | 3.17±0.02 | 8.75±0.4 | 13.59±0.13 | 2.95±1.31 | 8.47±0.15 | 12.47±0.24 | 8.62±7.33 |
| C20:2n-6 | 0.57±0.04 | 0.47±0.01 | n.d. | 0.59±0.04 | 0.51±0.01 | 0.25±0.1 | 0.63±0.03 | 0.51±0.01 | 0.27±0.23 |
| C22:0 | 0.27±0.02 | 0.24±0.01 | 0.75±0.23 | 0.28±0.03 | 0.27±0.01 | 0.45±0.63 | 0.27±0.01 | 0.28±0.01 | 0.27±0.1 |
| C20:3n-6 | 0.22±0.01 | 0.07±0 | 3.09±0.26 | 0.21±0.01 | 0.08±0 | 0.37±0.52 | 0.22±0 | 0.08±0 | 0.36±0.37 |
| C22:1n-9 | 0.74±0.02 | 0.06±0 | n.d. | 0.71±0.04 | 0.07±0 | 0.03±0.04 | 0.74±0.03 | 0.06±0 | 0.06±0.01 |
| C20:3n-3 | 0.22±0.01 | 0.14±0 | 4.78±2.8 | 0.21±0.03 | 0.15±0 | 2.81±0.61 | 0.23±0.02 | 0.15±0.01 | 1.89±2.52 |
| C20:4n-6 | 1.82±0.18 | 2.14±0.11 | n.d. | 1.8±0.19 | 1.78±1.2 | n.d. | 1.91±0.08 | 2.44±0.08 | 0.85±1.21 |
| C23:0 | 0.04±0.05 | 0.04±0.01 | n.d. | 0.04±0.04 | 0.02±0.03 | 0.09±0.13 | 0.19±0.11 | 0.07±0.02 | 0.19±0.27 |
| C20:4n-3 | 4.81±0.13 | 0.52±0.03 | n.d. | 4.45±0.27 | 0.57±0.01 | n.d. | 4.56±0.09 | 0.51±0.02 | 0.23±0.19 |
| C22:2n-6 | 0.1±0 | 0.04±0 | n.d. | 0.09±0 | 0.05±0 | 0.07±0.1 | 0.1±0.01 | 0.05±0 | 0.13±0.18 |
| C24:0 | 0.12±0.03 | 0.07±0 | 0.67±0.12 | 0.13±0.01 | 0.08±0 | 0.9±0.1 | 0.13±0.01 | 0.09±0 | 0.16±0.14 |
| C20:5n-3 | 7.99±0.39 | 7.73±0.16 | 2.85±0.46 | 7.89±0.47 | 7.45±0.11 | 1.68±0.19 | 8.31±0.24 | 7.43±0.1 | 4.51±3.56 |
| C24:1n-9 | 0.75±0.05 | 0.55±0.02 | n.d. | 0.78±0.04 | 0.67±0.01 | n.d. | 0.87±0.04 | 0.73±0.01 | 0.3±0.42 |
| C22:3n-3 | 0.29±0.02 | 0.15±0 | n.d. | 0.26±0.02 | 0.16±0 | n.d. | 0.29±0.02 | 0.15±0.01 | 0.1±0.01 |
| C22:4n-6 | n.d. | n.d. | n.d. | 0.02±0.03 | n.d. | n.d. | n.d. | n.d. | n.d. |
| C22:5n-3 | 2.39±0.11 | 0.43±0.01 | 0.17±0.07 | 2.18±0.12 | 0.43±0.01 | 0.03±0.05 | 2.19±0.07 | 0.38±0.02 | 0.21±0.13 |
| C22:6n-3 | 10.03±0.71 | 8.75±0.24 | 1.39±0.25 | 9.62±0.67 | 8.16±0.05 | 1.04±0.38 | 10.18±0.37 | 8.18±0.21 | 5.26±5.16 |
|  |  |  |  |  |  |  |  |  |  |

| Date | 4-5-2021 | 4-5-2021 | 4-5-2021 | 19-5-2021 | 19-5-2021 | 19-5-2021 | 8-6-2021 | 8-6-2021 | 8-6-2021 | 30-6-2021 |
| --- | --- | --- | --- | --- | --- | --- | --- | --- | --- | --- |
| Species | C. scutifer | E. graciloides | POM | C. scutifer | E. graciloides | POM | C. scutifer | E. graciloides | POM | POM |
| Sub samples | 3 | 3 | 2 | 3 | 3 | 1 | 3 | 3 | 2 | 1 |
| Total_lipids | 423.93±23.03 | 382.73±15.75 | NaN±NaN | 413.45±28.07 | 382.33±21.21 | NaN±NaN | 317.8±31.8 | 384.16±11.33 | NaN±NaN | NaN±NaN |
| C14:0 | 10.29±0.22 | 10.84±0.12 | 4.46±0.54 | 9.68±0.24 | 9.31±0.85 | 5.75±3.43 | 9.02±0.78 | 10.63±0.45 | 8.08±0.14 | 10.38 |
| iso-15:0 | 1.7±0.04 | 1.61±0.07 | 1.45±0.13 | 1.71±0.19 | 1.55±0.1 | 1.2±0.69 | 1.79±0.43 | 1.34±0.17 | 1.03±0.18 | 1.37 |
| anteiso-15:0 | 0.9±0.02 | 0.93±0.02 | 0.43±0.12 | 0.84±0.04 | 0.85±0 | 0.4±0.18 | 0.77±0.03 | 0.76±0.01 | 0.82±0.32 | 0.73 |
| C14:1n-5 | 0.09±0.01 | 0.08±0 | 0.04±0.05 | 0.09±0.01 | 0.06±0.01 | 0.11±0.04 | 0.08±0.01 | 0.06±0 | 0.13±0.01 | 0.2 |
| C15:0 | 0.87±0.03 | 0.68±0.01 | 0.51±0.23 | 0.89±0.03 | 0.67±0.01 | 0.48±0.1 | 0.7±0.05 | 0.6±0.03 | 1.06±0.59 | 0.75 |
| iso-16:0 | 0.84±0.17 | 0.65±0.02 | 0.1±0.14 | 0.87±0.1 | 0.52±0.12 | 0.08±0.11 | 0.6±0.05 | 0.47±0.08 | 0.25±0.08 | 0.17 |
| C15:1n-5 | 0.02±0.02 | n.d. | n.d. | 0.01±0.03 | n.d. | n.d. | 0.05±0.04 | 0.11±0.09 | n.d. | n.d. |
| C16:0 | 15.86±0.47 | 15.07±0.3 | 19.43±0.07 | 17.06±0.79 | 15.5±0.33 | 19.43±2.85 | 18.54±2.36 | 14.79±1.22 | 18.95±2.05 | 22.07 |
| iso-17:0 | 0.38±0.02 | 0.38±0 | 0.89±0.14 | 0.36±0.03 | 0.36±0.01 | 0.8±0.22 | 0.27±0.04 | 0.26±0.02 | 0.62±0.15 | 0.76 |
| C16:1n-9 | 0.62±0.11 | 0.43±0.05 | 0.66±0.17 | 0.77±0.25 | 0.5±0.14 | 0.67±0.12 | 1.1±0.56 | 0.51±0.16 | 2.23±1.76 | 0.64 |
| C16:1n-7 | 4.85±0.2 | 5.28±0.07 | 5.24±1.6 | 4.89±0.05 | 5.3±0.2 | 5.8±0.95 | 4.18±0.75 | 4.7±0.14 | 7.15±0.45 | 11.84 |
| C17:0 | 0.44±0.01 | 0.43±0.02 | 0.44±0.03 | 0.46±0.03 | 0.47±0.01 | 0.43±0.15 | 0.43±0.01 | 0.4±0.03 | 0.47±0.2 | 0.31 |
| 9,10D16 | n.d. | n.d. | n.d. | n.d. | n.d. | n.d. | n.d. | n.d. | 0.17±0.24 | n.d. |
| C17:1n-7 | 0.04±0.01 | 0.02±0 | 0±0 | 0.05±0.02 | 0.03±0.03 | 0±0 | 0.25±0.26 | 0.12±0.07 | n.d. | n.d. |
| C18:0 | 5.27±0.92 | 3.66±0.47 | 34.94±0.63 | 6.49±1.19 | 4.19±0.75 | 33.06±17.7 | 8.24±3.08 | 4.06±1.15 | 17.68±6.26 | 11.43 |
| C18:1n-9trans | 0.18±0.01 | 0.18±0.02 | n.d. | 0.22±0.01 | 0.18±0.02 | 0.08±0.11 | 0.17±0.05 | 0.13±0.02 | 0.12±0.07 | 0.12 |
| C18:1n-12 | 0.01±0.01 | 0.03±0.03 | n.d. | n.d. | 0.06±0.05 | 0.08±0.11 | 0.09±0.07 | 0.01±0.02 | 0.76±1.08 | n.d. |
| C18:1n-9cis | 4.09±0.18 | 5.81±0.09 | 2.92±0.5 | 4.04±0.13 | 5.85±0.2 | 3.24±1.12 | 4.28±0.5 | 5.81±0.44 | 7.19±1.62 | 7 |
| C18:1n-7 | 2.09±0.03 | 1.99±0.04 | 1.25±0.21 | 1.98±0.25 | 1.95±0.1 | 1.18±0.24 | 1.74±0.33 | 1.62±0.19 | 1.39±0.12 | 1.58 |
| C18:1n-6 | 0.1±0.01 | 0.07±0.02 | 0±0 | 0.14±0.07 | 0.14±0.08 | 0.61±0.87 | 0.8±0.64 | 0.28±0.19 | 0.3±0.43 | 0.85 |
| C19:0 | 0.43±0.04 | 0.25±0.03 | 0.62±0.12 | 0.57±0.15 | 0.28±0.05 | 0.63±0.19 | 0.81±0.29 | 0.46±0.1 | 0.67±0.02 | 0.87 |
| C18:2n-6trans | 0.01±0.01 | 0.01±0.01 | n.d. | 0.01±0.02 | 0.01±0.01 | n.d. | n.d. | n.d. | 0.12±0.17 | n.d. |
| 9,10D18 | 0.08±0.01 | 0.1±0.01 | n.d. | 0.11±0.02 | 0.12±0.01 | n.d. | 0.11±0.02 | 0.16±0.01 | n.d. | n.d. |
| C18:2n-6cis (LIN) | 4.45±0.14 | 6.95±0.09 | 1.91±0.69 | 4.39±0.13 | 6.77±0.24 | 2.11±0.72 | 4.04±0.59 | 6.1±0.32 | 4.51±0.06 | 4.19 |
| C20:0 | 0.51±0.01 | 0.4±0.02 | 0.49±0.07 | 0.55±0.01 | 0.4±0.02 | 0.47±0.03 | 0.45±0.01 | 0.3±0.02 | 0.45±0.04 | 0.44 |
| C18:3n-6 | 0.29±0.01 | 0.64±0.02 | 0.22±0.04 | 0.29±0.01 | 0.61±0.02 | 0.27±0.12 | 0.25±0.04 | 0.52±0.02 | 0.31±0 | 0.42 |
| C20:1n-9 | 0±0 | 0±0 | 2.83±0.87 | 0±0 | 0±0 | 3.53±1.03 | 0±0 | 0±0 | 5.6±0.73 | 6.03 |
| C18:3n-3 (ALA) | 7.18±0.18 | 9.98±0.14 | n.d. | 6.85±0.47 | 9.68±0.07 | n.d. | 6.92±0.25 | 9.48±0.25 | n.d. | n.d. |
| C21:0 | 0.09±0.01 | 0.04±0.01 | n.d. | 0.07±0.06 | 0.03±0.01 | n.d. | 0.06±0.01 | 0.02±0.03 | n.d. | n.d. |
| C18:4n-3 | 7.91±0.27 | 11.83±0.26 | 6.46±0.6 | 6.98±0.38 | 11.76±0.16 | 6.21±1.9 | 6.53±1.04 | 14.07±0.61 | 8.35±1.2 | 6.86 |
| C20:2n-6 | 0.63±0.03 | 0.52±0.01 | 0.06±0.09 | 0.62±0.03 | 0.52±0.03 | 0.03±0.04 | 0.5±0.09 | 0.44±0.03 | 0.06±0.08 | n.d. |
| C22:0 | 0.27±0 | 0.27±0 | 3.18±4.5 | 0.28±0.02 | 0.28±0.01 | 1.9±2.46 | 0.24±0.02 | 0.2±0.01 | 0.17±0.24 | 0.21 |
| C20:3n-6 | 0.22±0 | 0.11±0.03 | 0.38±0.54 | 0.24±0.03 | 0.08±0 | 0.57±0.16 | 0.18±0.02 | 0.09±0.02 | 0.13±0.18 | 0.39 |
| C22:1n-9 | 0.72±0.05 | 0.08±0.01 | n.d. | 0.69±0.07 | 0.06±0.01 | 0±0 | 0.59±0.13 | 0.04±0.01 | 0.02±0.02 | n.d. |
| C20:3n-3 | 0.23±0.01 | 0.14±0 | 3.27±0.47 | 0.29±0.03 | 0.14±0.01 | 3.21±1.62 | 0.27±0.02 | 0.13±0.02 | 2.17±0.26 | 2.57 |
| C20:4n-6 | 1.88±0.1 | 2.52±0.03 | n.d. | 1.73±0.06 | 2.53±0.15 | n.d. | 1.33±0.38 | 2.02±0.18 | n.d. | n.d. |
| C23:0 | 0.28±0.19 | 0.08±0.11 | n.d. | 0.46±0.37 | 0.19±0.16 | n.d. | 0.62±0.54 | 0.26±0.2 | 0.06±0.08 | n.d. |
| C20:4n-3 | 4.35±0.18 | 0.56±0 | 0.18±0.25 | 3.9±0.26 | 0.5±0.03 | 0.06±0.09 | 2.74±0.55 | 0.41±0.03 | 0.23±0.02 | n.d. |
| C22:2n-6 | 0.1±0.01 | 0.05±0 | n.d. | 0.12±0 | 0.05±0.01 | n.d. | 0.1±0.02 | 0.02±0.03 | 0.03±0 | n.d. |
| C24:0 | 0.13±0.01 | 0.1±0.01 | 0.3±0.15 | 0.15±0.02 | 0.1±0.01 | 0.26±0.02 | 0.13±0.02 | 0.06±0.01 | 0.51±0.23 | 0.32 |
| C20:5n-3 | 8.33±0.23 | 7.55±0.21 | 2.98±1.08 | 8.08±0.22 | 7.95±0.33 | 3.11±0.57 | 7.78±1.24 | 7.66±0.48 | 4.1±0.54 | 4.1 |
| C24:1n-9 | 0.87±0.05 | 0.77±0.03 | n.d. | 0.96±0.09 | 0.75±0.05 | n.d. | 0.94±0.17 | 0.65±0.05 | n.d. | n.d. |
| C22:3n-3 | 0.27±0.01 | 0.15±0.01 | n.d. | 0.25±0.05 | 0.14±0.01 | n.d. | 0.17±0.03 | 0.11±0.03 | n.d. | n.d. |
| C22:4n-6 | n.d. | n.d. | n.d. | n.d. | 0.01±0.01 | n.d. | 0.03±0.02 | n.d. | 0.02±0.03 | n.d. |
| C22:5n-3 | 1.98±0.14 | 0.43±0.01 | 0.17±0.05 | 1.85±0.15 | 0.36±0.02 | 0.04±0.06 | 1.31±0.27 | 0.33±0.02 | 0.16±0.01 | 0.14 |
| C22:6n-3 | 10.17±0.28 | 8.3±0.36 | 4.18±1.28 | 10±0.68 | 9.15±0.34 | 4.23±2.63 | 10.81±1.5 | 9.8±0.78 | 3.93±0.52 | 3.24 |
